# Systematic exploration of multiple drug binding sites

**DOI:** 10.1186/s13321-017-0255-6

**Published:** 2017-12-28

**Authors:** Mónika Bálint, Norbert Jeszenői, István Horváth, David van der Spoel, Csaba Hetényi

**Affiliations:** 1 Department of Pharmacology and Pharmacotherapy, Medical School, University of Pécs, Szigeti út 12, Pécs, 7624 Hungary; 2 Department of Biochemistry, Eötvös Loránd University, Pázmány Péter sétány 1/C, Budapest, 1117 Hungary; 3 MTA NAP-B Molecular Neuroendocrinology Group, Institute of Physiology, Szentágothai Research Center, Center for Neuroscience, University of Pécs, Szigeti út 12, Pecs, 7624 Hungary; 4 Chemistry Doctoral School, University of Szeged, Dugonics tér 13, Szeged, 6720 Hungary; 5 Uppsala Center for Computational Chemistry, Science for Life Laboratory, Department of Cell and Molecular Biology, University of Uppsala, Box 596, 75124 Uppsala, Sweden

**Keywords:** Peptide, Search, Pocket, Pharmacodynamics, Water, Interaction, Structure, Complex, Dissociation, Flexibility

## Abstract

**Background:**

Targets with multiple (prerequisite or allosteric) binding sites have an increasing importance in drug design. Experimental determination of atomic resolution structures of ligands weakly bound to multiple binding sites is often challenging. Blind docking has been widely used for fast mapping of the entire target surface for multiple binding sites. Reliability of blind docking is limited by approximations of hydration models, simplified handling of molecular flexibility, and imperfect search algorithms.

**Results:**

To overcome such limitations, the present study introduces Wrap ‘n’ Shake (WnS), an atomic resolution method that systematically “wraps” the entire target into a monolayer of ligand molecules. Functional binding sites are extracted by a rapid molecular dynamics shaker. WnS is tested on biologically important systems such as mitogen-activated protein, tyrosine-protein kinases, key players of cellular signaling, and farnesyl pyrophosphate synthase, a target of antitumor agents.
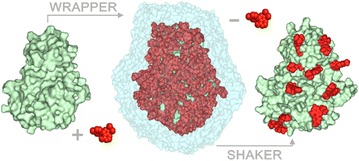

**Electronic supplementary material:**

The online version of this article (10.1186/s13321-017-0255-6) contains supplementary material, which is available to authorized users.

## Background

Molecular docking complements experimental structure determination and it has become a standard tool of drug discovery for the determination of protein–ligand complex structures [1]. The technique in practice is a compromise between computational cost and accuracy. Its high speed necessitates the use of severe approximations such as (i) restriction of the search space to the surroundings of the binding site, (ii) no or inadequate explicit hydration of the ligand-target interface, (iii) partial or complete neglect of target flexibility [2–5] during ligand binding, (iv) and non-deterministic search algorithms [1, 6] based on random number generation. Approximations i–iv seriously limit the applicability of docking methods for the following reasons. Restriction of the search to a primary binding site requires knowledge of its location and also neglects multiple sites such as allosteric ones [7, 8]. Water molecules often play a role in ligand binding [9–11] and ignoring interfacial water positions during docking may drive the ligands into pockets which are or should be filled with water molecules, resulting in incorrectly docked ligand poses [12]. Potential water release is also important during ligand binding especially through its entropic contributions [13, 14]. Neglecting or limiting the flexibility of target molecules is obviously incorrect at binding situations with induced fit [15]. Eventuality of random number generation in search engines such as Monte-Carlo or genetic algorithms [1, 5, 6] is a natural barrier of the reproducibility and reliability of the results.

The blind docking (BD) approach was introduced [16, 17] to extend the docking search to the entire target surface. In BD, previous knowledge and restriction of the search to a primary binding site are not necessary, and therefore, it can be used in search of multiple binding sites, as well. Indeed, BD has gained popularity [18–20] and has been used for finding allosteric [21–23], or multiple [24–28] binding sites. Thus, BD addresses the above first challenge and performs a global search instead of a focused one at an increased computational cost. However, approximations ii–iv cannot be remediated as simply as the first one. Promising approaches using explicit water molecules in the binding pocket [10] (approximation ii) and treating target flexibility (approximation iii) have been reported for focused docking [29]. However, such approaches have not been implemented in conjunction with solving the global search problem of BD on the entire target surface. Statistical evaluation of multiple docking trials has been shown to increase reproducibility of a BD search [17]; by using multiple randomized (approximation iv) initial ligand positions. Thus, it has become common to perform several docking trials with different initial positions in a BD search to ensure that the largest possible part of the target surface is scanned. However, even such a statistical evaluation cannot guarantee systematic and reproducible exploration of the entire target surface during BD.

Molecular dynamics (MD) simulations have an increasing impact on drug development [30–32]. A series of pioneering studies have reported the use of MD for tracking the ligand binding process [33–37], at atomic resolution. MD calculations also allow the use of explicit water molecules and flexible targets overcoming the above limitations from approximations ii and iii [38–40] potentially opening a new avenue for improvement of BD. MD simulations typically use random starting conformations for the ligands, likewise to BD. Generally, long MD calculation times are required for successful navigation of the ligand into the binding site such that the computational time necessary for accurate docking of a ligand may be prohibitive in practice. Pocket search methods were also developed, exploiting the above-mentioned advantages of MD [41]. A recent review [30] also concludes that “Improper preparation of the initial structure or insufficient equilibration of the initial structure(s) can impact the quality of the MD results”. The present study is aimed at overcoming the above uncertainties of present fast BD and molecular dynamics techniques, by combination of their advantages into a new strategy. Test applications are presented with successful identification of multiple binding sites on biologically important systems such as MAP and tyrosine-protein kinases, key players of cellular signaling as well as farnesyl pyrophosphate synthase, a target of antitumor agents.

## Algorithm

**Fig. 1:**
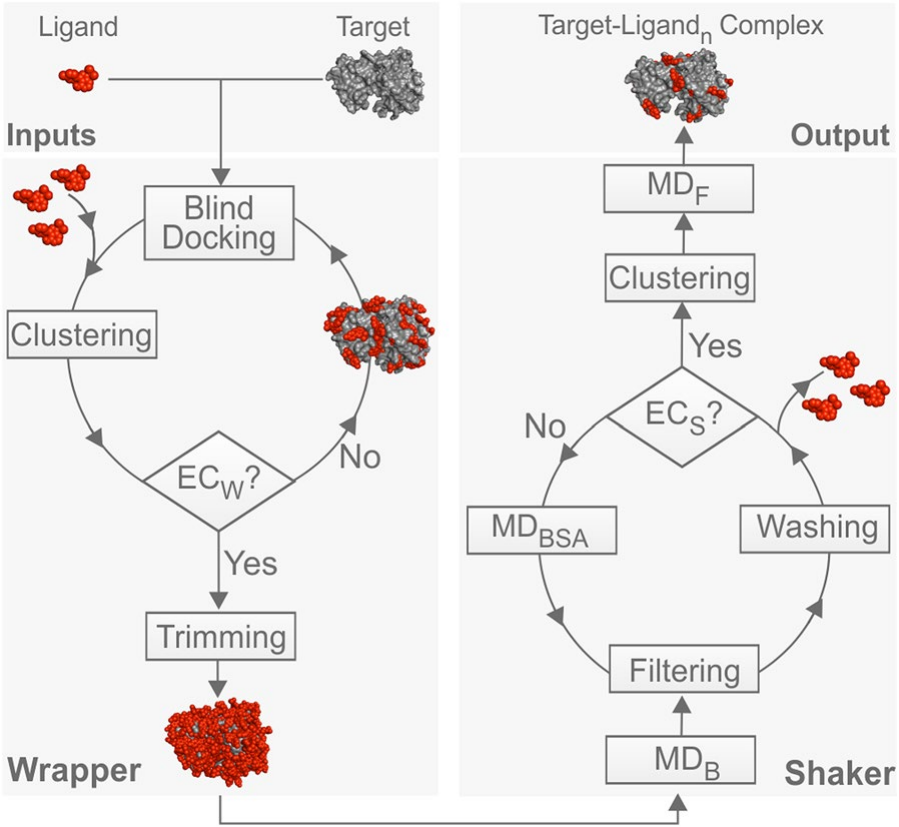
Wrap ‘n’ Shake flowchart featuring the main steps of the method. A quick overview is also presented in Additional file 1: Supporting Movie 1

Wrap ‘n’ Shake (WnS) is a new method composed of consecutive algorithms, the Wrapper and the Shaker (Fig. 1, Additional file 1: Supporting Movie 1) offering a systematic search for multiple binding sites and modes. WnS works in synergy with popular open source program packages AutoDock 4.2.3 [29] and GROMACS 5.0.2 [42].

### Wrapper

**Fig. 2:**
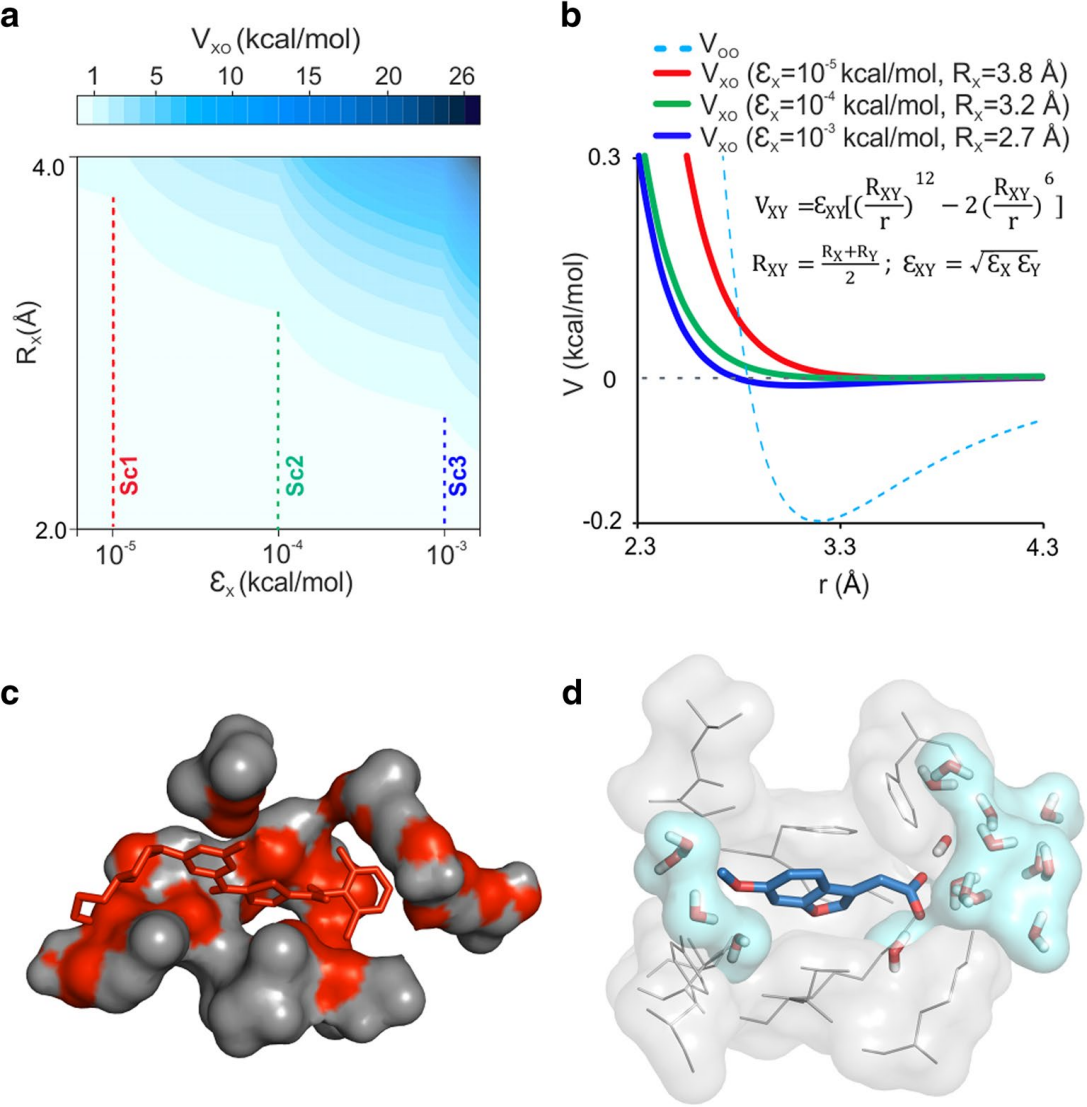
Systematic calibration of ε_X_ and R_X_. **a** A section of the V_XO_(r, ε_X_, R_X_) LJ potential function at r = 2 Å. Scenarios Sc1-Sc3 are shown with the magnitude of the R_X_ values corresponding to a short range repulsion of V_XO_ ≈ 1 kcal/mol (dashed lines) **b** V_XO_ LJ potential functions of scenarios Sc1-Sc3. V_OO_ of an oxygen atom pair is also shown for comparison. **c** An example of excluded atoms X (red, Cycle 1, Rank 2, System 9). Docked ligand conformation, is presented with sticks and the binding pocket is shown as surface. **d** Ligand of System 2 (dark blue sticks) bound to its target farnesyl pyrophosphate synthase (grey lines and surface) after Shaker (MD_F_ step). Explicit water molecules surrounding the ligand within 7 Å are shown with sticks and light blue surface

Wrapper performs several fast BD cycles by AutoDock 4.2, and AutoGrid 4.2 [29] and systematically covers the entire surface of the target with a monolayer of ligand copies (Fig. 1). Each BD cycle is performed as described in Additional file 2: Table S1, and results in 100 docked ligand copies, which are ordered by their interaction energies with the target, and structurally clustered. To achieve a ligand monolayer, the ligand–ligand interactions are minimized through implementation of a weak repulsion between the docked ligand copies, and therefore blocking the formation of ligand aggregates (Additional file 2: Table S2). At the same time, target-ligand interactions are maximized (Additional file 2: Table S3) to ensure that the largest possible numbers of new ligand copies are placed on the surface in an actual BD cycle. The initial experiments (Additional file 2: Table S2) also showed that introduction of a weak repulsion is essential to avoid erroneous ligand geometries clashing with target atoms. Such unwanted clashes (Additional file 2: Table S2) were obtained if intermolecular electrostatic (E_Coulomb_) and van der Waals (E_LJ_, Eq. 1) interaction energy terms were simply switched off at the ligand atoms. Notably, calculation of total target-ligand intermolecular interaction energy (E_inter_) in AutoDock 4.2 is based on the scaled E_Coulomb_ and E_LJ_ terms of the Amber96 force field [43], and an estimate for de-solvation free energy changes (ΔG_sol_, Eq. 1). E_LJ_ is the sum of Lennard-Jones potential energy values (V, Fig. 2) calculated for all target-ligand atom pairs.

1 $${\text{E}}_{\rm inter} = {\text{E}}_{\rm Coulomb} + {\text{E}}_{\rm LJ} + {\Delta\text{G}}_{\rm sol} .$$

Finally, instead of the above-mentioned, oversimplified attempt of switching off all intermolecular terms of E_inter_ we elaborated a new protocol which produced the desired ligand monolayer by introduction of an excluded atom type (X). In this protocol, all ligand copies docked in a cycle and their surrounding target atoms are excluded from the next cycle (red in Fig. 2c), and only the unbound target surface (grey) is used for a next BD cycle. The neighboring target atoms are selected by an interface tolerance of 3.5 Å, the maximal distance between a target heavy atom and the closest docked ligand heavy atom. The above exclusion of certain atoms during docking is physically achieved by modification of the non-bonding terms of E_inter_. For this, the new atom type X is assigned for excluded atoms (red in Fig. 2c) by a C program Wrp developed for this study. Wrp switches off E_Coulomb_ by setting the partial charge of X to zero and also assigns new LJ parameters.

The new LJ parameters were fine-tuned for atom type X in order to produce the necessary weak repulsions described above. Briefly, the LJ parameters of X were calibrated considering the pairwise LJ potential between atom types X and Y (V_XY_) at three common atom types (Y=O, C and H). A systematic search of both equilibrium potential well-depth (ε_X_, Fig. 2a) and inter-nuclear distance (R_X_) was conducted. Numerous docking runs were performed to evaluate the effect of the selected LJ parameters. A pre-defined value of r = 2 Å (ca. a covalent bond + 0.5 Å) was used as a minimal distance where short-range repulsion should act at a desired maximal value not exceeding a V_XY_≈1 kcal/mol. Three scenarios (Sc1-Sc3) were evaluated as shown in the r = 2 Å section of V_XO_ (r, ε_X_, R_X_) function (Fig. 2a) calculated for the XO atom type pair. Sc2 (green line, Fig. 2a, b) was identified as an optimal scenario with an ε_X_ = 10^−4^ kcal/mol and an R_X_ of 3.2 Å (approximate distance between heavy atoms in an H-bond). In this case, available target surface is optimally used without large ligand-free zones in the monolayer. A short-range repulsion was achieved (green line in Fig. 2b) with a zero value beyond the repulsion zone. If R_X_ was too large (Sc1, red in Fig. 2a, b) then the repulsion zone around the docked ligand copies would also increase with a V_XO_ curve shifted to the right if compared to the green curve of Sc2 (Fig. 2b) resulting in large ligand-free zones, i.e. a non-optimal arrangement of the ligand copies in the monolayer. Importantly, the repulsion zone in the optimal V_XO_ curve of Sc2 starts at lower distances (r) than in the V_OO_ curve. V_OO_ is shifted to the right of the red curve (Sc1), which would result in even larger ligand-free regions than Sc1. Thus, using only a repulsion term of V_OO_ would have not been adequate for exclusions of atoms in wrapping. On the other hand, if R_X_ was too small (Sc3, blue in Fig. 2a, b), then unwanted attractive effects such as aggregation between docked ligand copies would still happen similar to Trial 1, in Additional file 2: Table S2. Accordingly, in Sc3 the corresponding blue curve is shifted to the left from the green Sc2 curve (Fig. 2b). The same procedure was repeated for atom types Y = C and H and an average R_X_ value of 3.6 Å was concluded (Additional file 2: Table S3) and used in Wrapper along with the above ε_X_ = 10^−4^ kcal/mol.

These calibrated LJ parameters of X allowed elimination of the above-mentioned unwanted interactions between the newly docked ligand copies and the already filled binding pockets (Fig. 2c). As the introduced repulsive potential acts on a short range, the ligands can still dock to other, unbound parts of the target surface. The new atom type and parameters also maximize target-ligand interactions adding the maximal number of ligand copies to the mono-layer during a BD cycle.

Wrapper cycles are terminated by either the drop of uncovered surface area of the target below one percent of its total (ligand-free, initial) surface area, or positive target-ligand interaction energy in every cluster representative (EC_W_ in Fig. 1). As a last step, a trimming is performed to remove all ligand copies situated more than 3.5 Å from the target. Wrapper results in a target wrapped in N ligand copies (target-ligand_N_ complex) provided as a single Protein Databank (PDB) file. Wrapper is implemented in a new open source package WnS as shell scripts and a C program Wrp available for download together with a User’s Manual at www.wnsdock.xyz.

### Shaker

Shaker selects functional binding sites by removing non-specific, loosely bound ligand copies from the target surface. The target-ligand_N_ complex is placed in a box filled with water and subjected to MD simulations in consecutive cycles. The cycles are performed until a 75% of the ligand copies are eliminated (Exit Criterion of Shaker, EC_S_ Fig. 1). In each Shaker cycle, distance and energy metrics are calculated describing target-ligand interactions at each time step (frame) of a trajectory. The metrics include the closest distances between the target and the ligand as well as E_LJ_, calculated using Amber parameters. Based on these metrics, filtering (Additional file 2: Table S4) and subsequent removal of the corresponding ligand co-ordinates (Washing, Fig. 1) are applied to exclude ligand positions dissociated from their starting binding positions. The filtering involves two distance-based steps and two final steps based on E_LJ_.

Before the first cycle a 5-ns target backbone-restrained MD (MD_B_) is used to grossly shake off the weakly bound ligands. In cases where this initial MD is not enough to reach the required EC_S_ (Additional file 2: Table S1 and Additional file 2: Table S7), multiple cycles with 20-ns simulated annealing (MD_BSA_) simulations are performed, using position restraints on the target backbone atoms. Depending on the molecular weight (MW, Table 1) of the ligands, SA was done, using two temperature protocols, up to 50 °C (MW ≤ 300) or 80 °C (MW ≥ 300). High temperature in SA accelerated the dissociation process as expected. After MD_BSA_ cycles, a clustering and ranking step is performed, using the last frames of the remaining ligands. A refinement of 20-ns MD with full protein flexibility (MD_F_) is also performed on every target-ligand complex resulted after clustering (Additional file 2: Table S7 and Additional file 2: Table S8). The Shaker protocol (Additional file 2: Table S9) was formulated during multiple trials (Additional file 2: Tables S5 and S6) and results in a final solution structure of a target-ligand_n_ complex, where n is the total number of final cluster representatives.

**Table 1:** Test systems

#	PDB ID^a^	Target	Ligand	MW^b^
1	3ptb	bovine β-trypsin	benzamidine	120
2	3n3 l	farnesyl pyrophosphate synthase	(6-methoxy-1-benzofuran-3-yl) acetic acid (MS0)	206
3a	3hvc	mitogen-activated protein kinase	4-[3-(4-fluorophenyl)-1 h-pyrazol-4-yl]pyridine (GG5)	239
3b	4f9w	mitogen-activated protein kinase	4-[3-(4-fluorophenyl)-1 h-pyrazol-4-yl]pyridine (GG5)	239
4	3cpa	carboxy-peptidase	GY	256
5	1qcf	haematopoetic cell kinase (HCK)	1-ter-butyl-3-p-tolyl-1 h-pyrazolo[3,4-d]pyrimidin- 4-ylamine (PP1)	281
6	1h61	pentaerythritol tetranitrate reductase	Prednisone^®^	358
7	2bal	mitogen-activated protein kinase	[5-amino-1-(4- Fluorophenyl)-1H-Pyrazol-4- yl] [3-(piperidin-4-yloxy) phenyl]methanone	380
8	1hvy	thymidylate synthase	Ralitrexed^®^	459
9	3g5d	tyrosine-protein kinase Src	Dasatinib^®^	488
10	1be9	PDZ-domain	KQTSV	544

^a^PDB ID of the holo X-ray structure

^b^Molecular weight of the ligand

## Systems and test metrics

A diverse set of ten target-ligand systems were selected (Table 1) and prepared (Additional file 2: Table S1) as test cases of WnS. Challenging systems with multiple (prerequisite or allosteric) binding sites were included (Table 1). Our selection contains both small ligands and bulky, flexible ones. Apo protein structures were used as targets except System 8. In the case of System 5 another protein tyrosine-protein kinase was used as apo structure similar to a previous study [33].

Three standard metrics were used to quantify the results of tests. (1) root mean squared deviation (RMSD) measures structural precision of WnS results by comparison of atomic positions of ligand conformations produced by WnS and those of the crystallographic reference. Prior to calculation of RMSD, a structural alignment (Additional file 2: Table S10) was performed on the holo and apo target residues surrounding the ligand within 5 Å similarly to a previous work [33]. (2) Shaker Rate (SR = N/n) is a ratio of counts of the N ligand copies residing on the target surface (N) after Wrapper and the n final cluster representatives (n) produced by Shaker. The larger the SR, the more efficiently Shaker eliminated ligand copies from the target surface. (3) Rank serial number (#Rank) is calculated using relative ligand-target interaction energies corresponding to the docked ligand positions. WnS ranks docked ligand copies by their interaction energies with the target. The smaller the #Rank, the stronger the target-ligand interaction is at a ligand position. The #Rank of the docked ligand copy of the lowest RMSD is listed for all systems in Table 2. In the final rank lists, docked ligand copies with small RMSD, i.e. close to the crystallographic conformations should be preferably placed at the top of the rank lists, with small #Rank values.

**Table 2:** Results for the test systems

#	N^a^	CLS^b^	#Rank^c^		n^d^	SR^e^
			MD_BSA_	MD_F_		
1a	68	6	1	1	6	11
1b^g^	74	5	1	–	4	19
1c^g^	71	6	1	–	5	14
2	300	18	2	4	13	23
3a	222	46	3	4	21	11
3b	222	46	9	12	21	11
4 ^h^	155	12	1	1	8	19
5	143	25	2	1	12	12
6^i^	116	26	1	2	12	10
7	123	26	4	4	12	10
8	106	25	1	1	10	11
9^j^	92	23	2	1	10	9
10	49	11	2	1	4	12

^a^Total count of ligand copies after Wrapper

^b^Count of ligands surviving the Shaker, after MD_BSA_

^c^Rank serial number of the structure with the best RMSD value, after MD_BSA_ and after MD_F_

^d^Count of cluster representatives (final solutions) Shaker

^e^Shaker Rate

^f^Total computational time required for MD_B_, MD_BSA_ and MD_F_, as explained in Additional file 2: Table S12

^g^For System 1, WnS was performed with different seeds for data reproduction purposes

^h^Final clustering was done using van der Waals and Coulomb interactions due to interactions of zinc ion with the ligand

^i^Wrapper process was done, using the LJ interaction as a scoring function, instead of AD4 (Additional file 2: Table S13)

^j^Final clustering was done with 6 Å distance limit between clusters

## Results and discussion

### Association or dissociation?

**Fig. 3:**
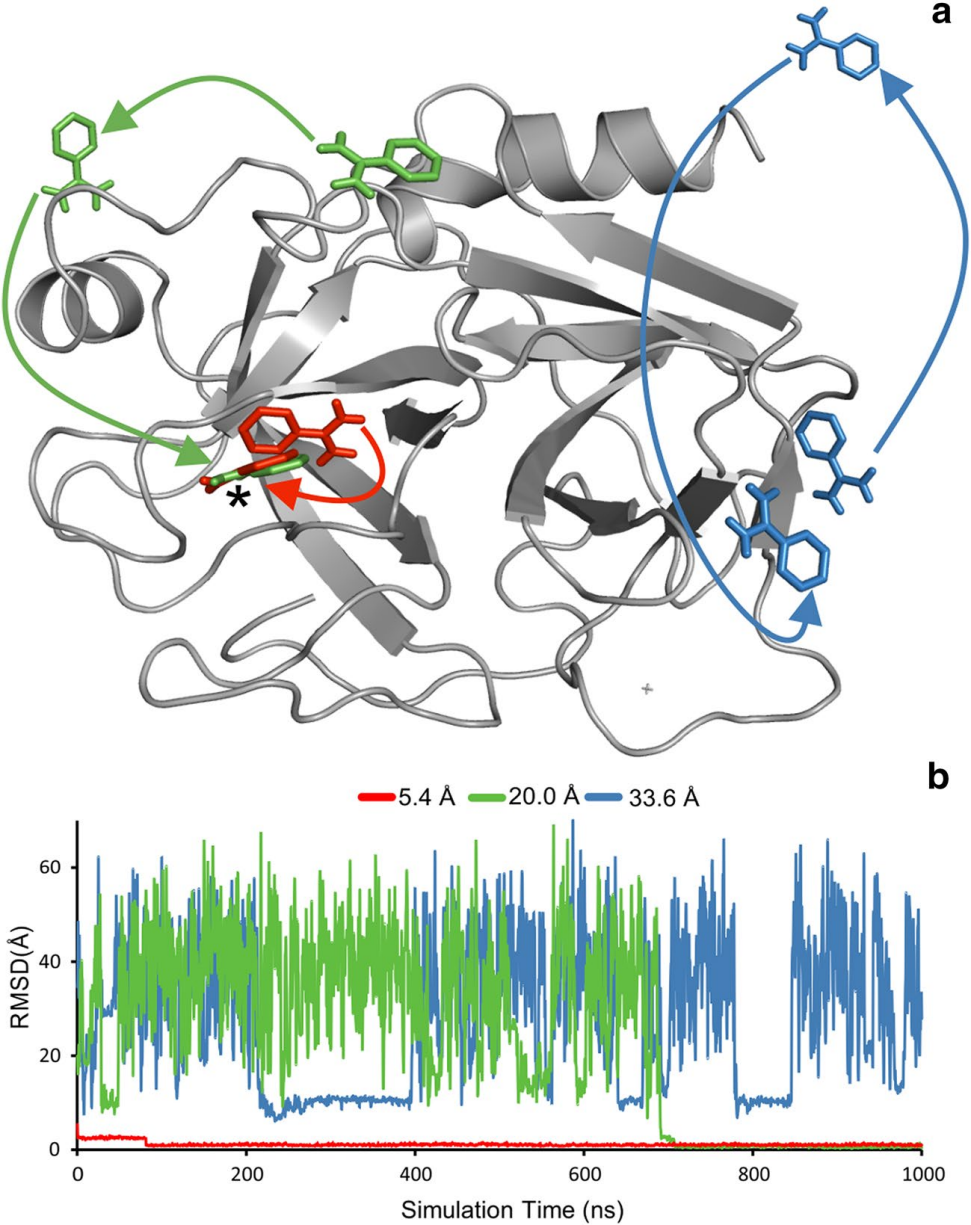
Pilot molecular dynamics simulations. Benzamidine ligand (sticks) started the MD simulations from three positions at different distances (as indicated in the legend) from the native binding site on the trypsin target (grey cartoon). Arrows in **a** point from starting (t = 0 ns) to final (t = 1000 ns) ligand positions. Only two of the three 1000 ns-long simulations with the closest starting position succeeded in finding the reference binding pose (*) known from the crystallographic structure (3ptb). **b** Time-dependence of root mean squared deviation (RMSD) of the ligand measured from its reference pose

Encouraged by results of pioneering MD studies [31, 33, 34], association of ligand benzamidine to bovine trypsin was followed in three MD simulations. Benzamidine is an easy case for docking and it has also been used in tests of recent approaches [44]. The present MD simulations were 1-µs-long and benzamidine was placed at three different starting positions (Fig. 3, Additional file 2: Table S11), at various distances (Fig. 3a) from the crystallographic binding site.

Analysis of the trajectories shows that the crystallographic binding position was found in two out of the three simulations after 81 and 690 ns simulation time (drop of red and green lines in Fig. 3b), respectively. In the 3rd case with the largest starting distance, 1 µs was not enough to dock to the native site by association (blue line). Thus, the usefulness of association MD runs for docking strongly depends on the starting ligand position even in the easy case of benzamidine. MD needs a simulation time comparable to the real association time of the ligand (Fig. 3b). This can be considerable, as migration of the ligand is hindered by friction in the surrounding water. Previous studies [33, 36, 45], have also reported simulations of several hundreds of nanoseconds for navigation of the ligand to the desired binding pocket.

All-in-all, the necessary time for successful docking by association MD depends on the actual starting position of the ligand, the size and shape of the target, ligand etc. To overcome such uncertainties on simulation length and still use the benefits of MD we elaborated a new strategy, the Wrap ‘n’ Shake (WnS, Fig. 1). Instead of simulating the association process, WnS is based on the dissociation of the ligand. Dissociation is fast and reproducible at binding sites of low stability.

### A systematic approach

Naturally, a dissociation approach requires a set of ligand copies bound to the target. Systematic mapping of all possible ligand positions (sites) cannot be guaranteed in a single BD cycle (Introduction) even if it contains hundreds of fast BD trials [17]. A truly systematic algorithm should completely wrap the entire surface of the target in a monolayer of copies of the ligand molecule. Our initial guess of such a Wrapper algorithm was based on a previous finding [17] that the coverage of the target can be increased with several, successive fast BD cycles where accumulated docked ligand copies from the previous cycle are considered as part of the target in the next cycle. However, additional experiments with such successive BD cycles showed that previously and newly docked ligand copies can easily form multi-layer aggregates with each-other instead of the target (Additional file 2: Table S2). The formation of such aggregates hinders wrapping of the target surface into the desired monolayer of ligand copies.

**Fig. 4:**
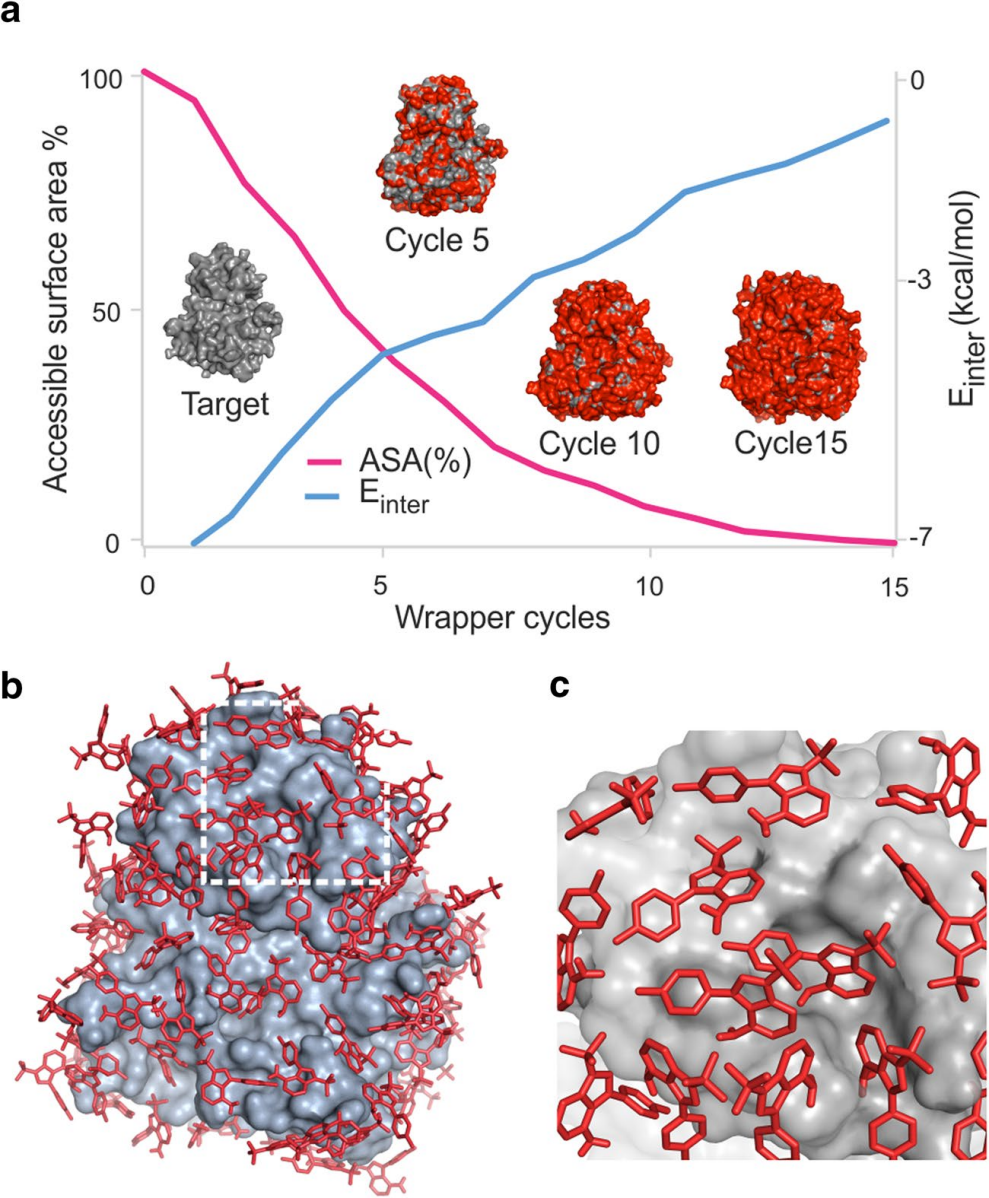
Wrapping tyrosine-protein kinase Src target into a mono-layer of ligand copies (System 5). **a** Unbound (ligand free) accessible surface area (ASA) of the target and the lowest E_inter_ of the cluster representatives in consecutive wrapping cycles. Target-ligand interaction energy (E_inter_) increases with increasing number of cycles finding strong binding sites in the first few cycles, before the final, saturation region. ASA finally decreases below 1%. Structural images show the wrapping of the target (grey surface) with ligands (red). **b** The monolayer arrangement of the ligands (red sticks) wrapping the entire target surface (grey) after the final cycles. **c** A close-up of a section of the monolayer showing that the ligand copies are evenly arranged without overlap

During the wrapping process, parts of the target surface already covered with ligand copies has to be excluded from interactions with ligand copies docked in a next BD cycle. This task is not trivial as potential functions of the docking force fields normally cannot distinguish between target sites unbound and covered with ligands. After extensive experimentation including an optimization of the force field (“Wrapper” section, Additional file 2: Table S3, Appendix 1) we arrived at a new algorithm called Wrapper (Figs. 2, 4). Wrapper performs a systematic coverage of the target surface in several, consecutive fast blind docking cycles (Fig. 4). The algorithm continuously monitors the status of coverage of target surface (Fig. 4a) and results in the desired monolayer of N ligand copies not interacting with each-other. Figure 4b shows an example of such a monolayer. Ligands constituting the monolayer have physically realistic arrangement (Fig. 4c), maximized interactions with the target and no contacts with each-other. Thus, the target is systematically and rapidly wrapped in a monolayer of N (Table 2) ligands.

**Fig. 5:**
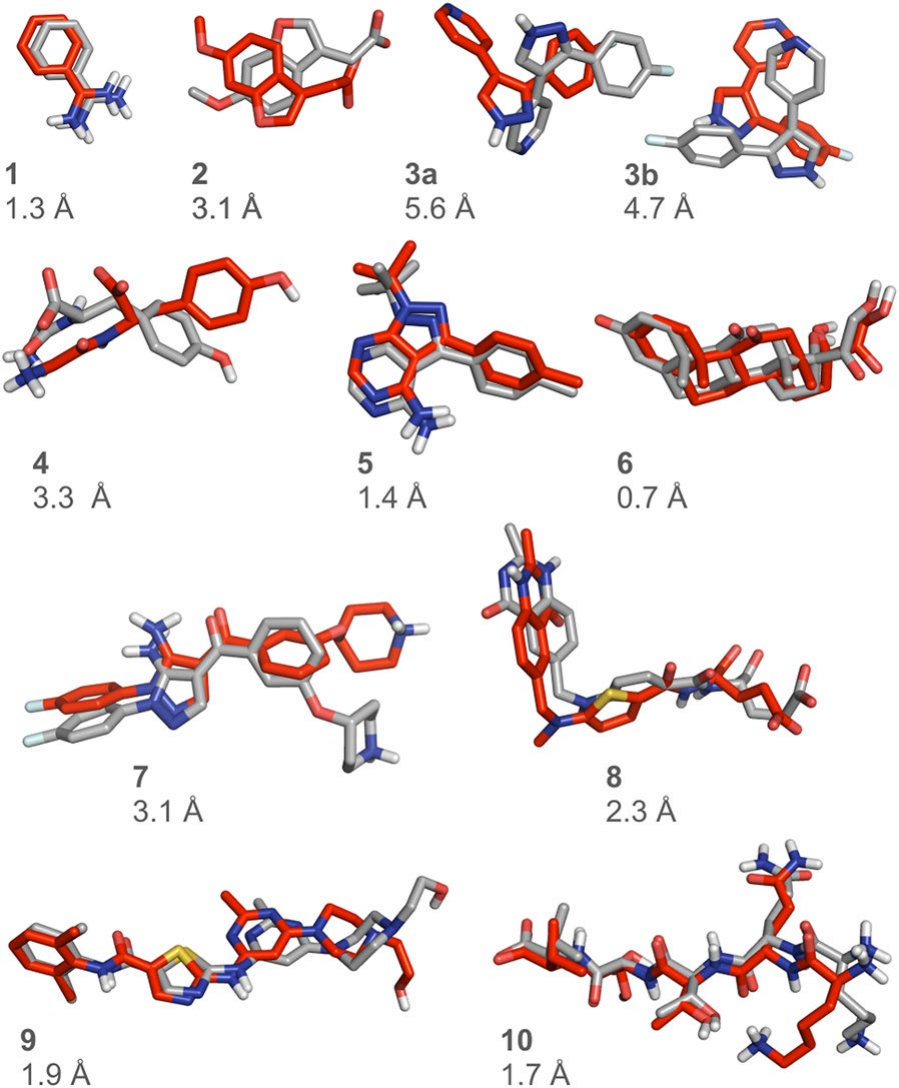
Structural fits quantified as root mean squared deviation (RMSD) with values given in Å. Ligand conformations after Shaker (grey) compared to the crystallographic references (red sticks). System# is bold

Having a realistic input geometry, the resulting target-ligand_N_ complex is transferred to the Shaker including MD simulation(s) with explicit water (“Shaker” section), filtering, and clustering steps. These steps eliminate ligands dissociated during MD and result in a strong binder at each pocket (Additional file 2: Table S7). Final results are shown in Table 2 using test metrics described in “Systems and test metrics”. Parameter SR characterizes efficiency of removal of loose binders. SR values of Table 2 indicate that a considerably large part of the weak binders were efficiently removed at all test systems beyond the default EC_S_ of 75% (SR = 4). Other important metrics are RMSD and #Rank. In most of the systems analyzed, ligand conformations with the lowest RMSD were placed into the first two ranks (Table 2, Fig. 5, and Additional file 2: Table S8). For stable ligand copies, good structural matches to the corresponding reference conformations (Fig. 5 and Additional file 2: Table S8), as well as low #Rank values (Table 2) were found. Fair results were obtained for challenging cases too (Systems 2 and 3). The somewhat lower rank in these cases may be explained by the relatively high B-factor of the ligands of these systems (Additional file 2: Table S1) suggesting an increased mobility and a less stable target-ligand interaction.

For example, B-factors of measured atomic positions of ligand MSo (System 2) vary in a range between 54 and 95 Å^2^ (Additional file 2: Table S1). During MD_F_ simulations we found that the RMSD varied between 2.5 and 5.1 Å (Additional file 2: Table S8), and a final #Rank of 4 and an RMSD of 3.1 Å were obtained. Considering the above high B-factor values, it is realistic to assume that ligand MSo adopts various conformations when bound to farnesyl phosphate synthase (System 2) including the one close to the assigned position found with an RMSD of 2.5 Å. This conformational variability of the bound MSo is probably due to its carboxylate group with the highest B-factor of 95 Å^2^. This group is hydrated by bulk water molecules, helping the dissociation of MSo from the target (Fig. 2d). At the same time, MD simulations with explicit water molecules also account for a hydrophobic, anchoring interaction between the benzofuran part of MSo (no waters present, Fig. 2d) and the target. This example shows the necessity of use of explicit water model during the shaking process in order to account for all, even antagonistic interactions.

In our pilot study (“Association or dissociation?” section) it was demonstrated that MD methods following the association pathways often need large amount of computational time and/or a fortunate starting conformation in order to find the primary site correctly for System 1. WnS yielded the correct solution for this system (Additional file 2: Table S8) in a 5-ns-long MD_B_ simulation which is at least one order of magnitude shorter than the lengthy association times discussed in “Association or dissociation?” section. Elimination of ligand excess (dissociation of ligand copies) (Tables S14 and S15) at an SR of 11 was facilitated by hydrogen bonding with explicit water molecules [46, 47]. Thermal motion of water molecules also contributed to fast “shake off” of the ligand copies especially in the cases of Systems with small ligands with the application of the simulated annealing protocol (MD_BSA_, see an SR of 23 in case of System 2 in Table 2).

### A case with a small ligand

**Fig. 6:**
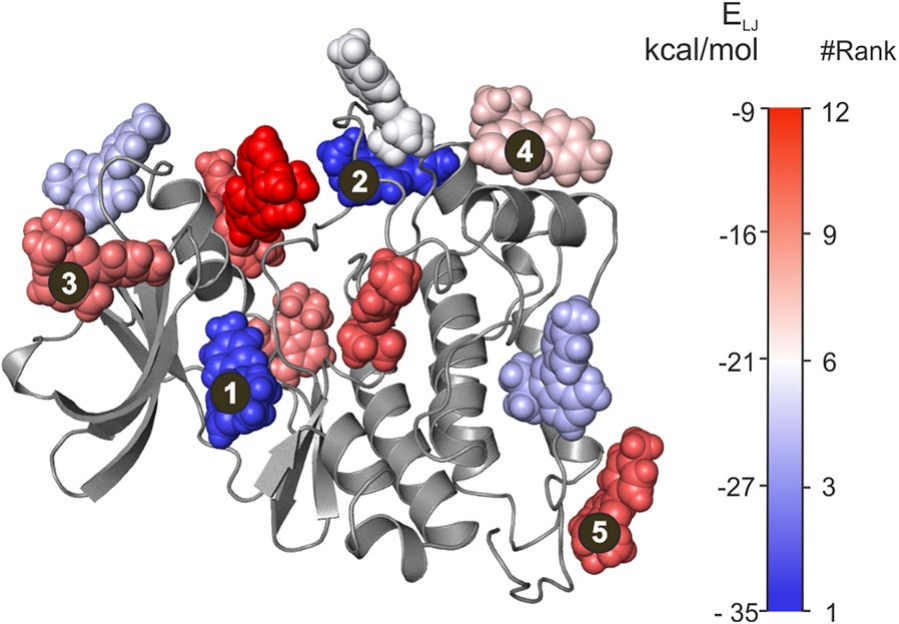
Haematopoetic cell kinase (HCK, System 5) with ligand copies remaining after Shaker. Ligand copies are colored by the calculated target-ligand interaction energy E, and the #Rank assigned. The previously reported pockets 1(ATP), 2(A-loop), 3(PIF site), 4(G-loop) and 5(MYR) are numbered by their increasing E_LJ_

WnS was tested on tyrosine protein kinase target with a pyrazolopyrimidine 1 ligand (PP1, System 5). Regulation of kinase activity is important in numerous human diseases [48, 49]. At the same time, this kinase is a challenging test target for WnS as it has multiple sites including an allosteric one identified in previous studies [50, 51]. The native, PP1 site was found (Fig. 5) at an excellent RMSD agreement (1.4 Å, Fig. 5) with the crystallographic position. Besides obtaining very good RMSD (Fig. 5), the #Rank was improved from second to first place (Table 2) during the final MD_F_ simulation (Additional file 2: Table S16). Apart from the primary site, our goal was to find other, prerequisite binding sites, as well. As described in a previous MD study [33], such sites correspond to poses on the binding pathway leading to the primary site. WnS found both low- and high energy prerequisite sites described previously [33] (Fig. 6). Besides structural matches, #Rank and the corresponding energy values are also comparable to the previous results. Notably, WnS can predict multiple binding sites beyond experimentally observable ones. These binding sites can be considered as prerequisite or allosteric binding sites. Previous MD results [33, 52] concluded, that finding prerequisite binding sites is a substantial advantage of the MD simulations.

### Cases with large ligands

**Fig. 7:**
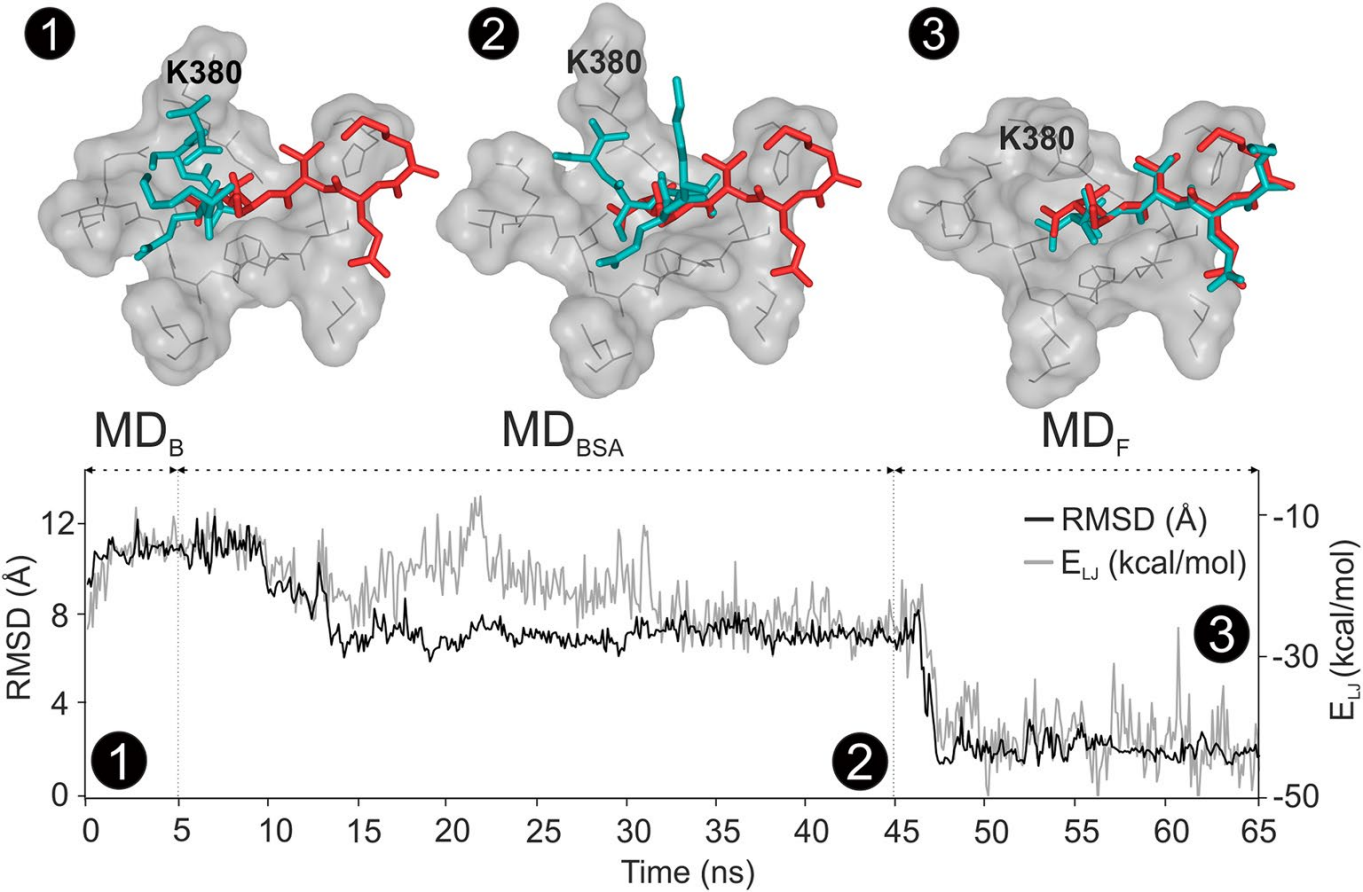
During Shaker, conformational changes of the pentapeptide KQTSV are observed, while remains bound to its pocket on the PDZ domain (System 10). Red sticks represent the native ligand conformation from PDB (1be9). Teal sticks represent ligand conformations at different Shaker stages starting with the conformation right after Wrapper (**1**), and continuing with conformation after MD_BSA_ (**2**), and after MD_F_ (**3**). The changes of target-ligand interaction energy (E_LJ_) and the RMSD during the MD stages in the Shaker protocol are plotted below the structural snapshots. See also Additional file 3: Supporting Movie 2 for further details of conformational changes

Tyrosine kinase also binds dasatinib (System 9), a bulky ligand, for which an SR of 9 was obtained (Table 2), after six simulated annealing cycles (Additional file 2: Table S12). The same four binding pockets were found for dasatinib as for the above PP1 (Additional file 2: Table S17). After the final MD_F_ step, local conformational refinement of dasatinib was observed, improving the RMSD from 2.3 to 1.9 Å. Similar to PP1, this could be partially explained by the role of the water molecules and the enhanced target motion during MD_BSA_. WnS was further tested on the challenging System 10 with a pentapeptide ligand with twenty-three flexible torsions. The correct binding position of the ligand was obtained after the MD_F_ stage of Shaker with an improvement of RMSD from 6.8 to 1.7 Å (Fig. 7, Additional file 3: Supporting Movie 2).

A re-ranking (Table 2) from Rank 2 to Rank 1 was also observed after MD_F_. For comparison, the wrapped target-ligand_N_ complex of System 10 was subjected directly to an MD_F_ simulation skipping the MD_B_ and MD_BSA_ steps of Shaker. In this case, an RMSD of 11.3 Å (Line 10b in Additional file 2: Table S8) was obtained which was worse than the RMSD obtained with the complete Shaker protocol (1.7 Å, Fig. 5). This demonstrates that both MD_B_ and MD_BSA_ steps of Shaker are necessary to find the correct position. After Wrapper, the pentapeptide was in a closed, cyclic conformation (Fig. 7, Snapshot 1). This unrealistic arrangement was opened up (Snapshots 2 and 3) by interacting water molecules. It can be also observed that limited protein flexibility during MD_B_ and MD_BSA_ allowed only moderate reduction of the ligand RMSD by improvement of the target-ligand interactions. Most of the RMSD and interaction energy improvement was achieved after MD_F_, and rearrangement of K380 inside the pocket was necessary, to improve the conformation of the simulated ligand (Fig. 7). All-in-all, MD steps including target flexibility have a significant influence on the results of WnS for large ligands. Introduction of MD_F_ considerably improved structural precision, in the above case studies of large ligands (Systems 9 and 10).

## Conclusions

In the present study, a systematic strategy, the Wrap ‘n’ Shake was introduced for exploration of multiple binding sites and modes of drugs on their macromolecular targets. Wrap ‘n’ Shake systematically wraps the target into a monolayer of ligand copies using a modified blind docking approach and selects stable positions by shaking off loose binders. The method offers a computationally feasible solution for the present problems of the field (Introduction). Wrapper requires only fast blind docking cycles with a program package such as AutoDock 4.2.3. The Shaker process is fairly short and can be performed by available MD packages. Shaker is further accelerated by simulated annealing and uses all benefits of explicit water model and target flexibility. Wrap ‘n’ Shake is suitable to study interactions of protein targets with even large peptide ligands. We have started the extension of the method towards protein ligands using a fragment-based approach with post hoc reconstruction of the ligand. In future applications, Wrap ‘n’ Shake could be also used for general pocket search, besides docking of individual ligands. We envision that Wrap ‘n’ Shake can become the tool of choice for systematic exploration of multiple binding sites and modes of ligands in drug design and structural biology.

## Additional files

**Additional file 1.** Supporting Movie 1 featuring the processes of Wrapper and Shaker in the case of System 5. The first part presents the results of 15 wrapping cycles. The second part contains MD_B_ and two MD_BSA_ cycles of Shaker. Final cluster representatives are the outputs of WnS. Additional refinement steps are shown in Supporting Movie 2 (Additional file 3).

**Additional file 2.** Supporting Tables S1–S17 and Appendix 1–4 with detailed methods and results.

**Additional file 3.** Supporting Movie 2 featuring conformational changes of pentapeptide KQTSV, bound to PDZ-domain (System 10) during 65 ns simulations performed Shaker. The binding pocket of KQTSV on the PDZ domain is presented with grey surface. The simulated and crystallographic reference structures of KQTSV are presented as teal and red sticks.

## Abbreviations

BD: blind docking
EC_W_: exit criterion of Wrapper
EC_S_: exit criterion of Shaker
E_inter_: intermolecular interaction energy
E_LJ_: Lennard-Jones potential
MD_B_: molecular dynamics with backbone position restraints
MD_BSA_: molecular dynamics with backbone position restraints and simulated annealing
MD_F_: molecular dynamics without restraints (flexible simulation
PDB: protein data bank
RMSD: root mean squared deviation
SR: Shaker Rate
WnS: Wrap ‘n’ Shake
