# Supplementary material for: Systematic exploration of multiple drug binding sites
Source: J Cheminform. 2017 Dec 28;9:65. doi: 10.1186/s13321-017-0255-6 (PMC5745209; doi:10.1186/s13321-017-0255-6)
Supplement: Supplementary file 2 — Additional file 2. Supporting Tables S1–S17 and Appendix 1–4 with detailed methods and results. [file 13321_2017_255_MOESM2_ESM.pdf]

*Supporting Information*

# Systematic exploration of multiple drug binding sites

Mónika Bálint, Norbert Jeszenői, István Horváth, David van der Spoel, Csaba Hetényi*

*Corresponding author.

## Table of contents

Table S1. Detailed General Methods .......... 4
Table S2. Wrapper: A pilot study .......... 8
Table S3. Wrapper: Development of force field parameters .......... 10
Table S4. Filters of the Shaker algorithm .......... 13
Table S5. Shaker: Pilot study 1: $MD_B$ .......... 15
Table S6. Shaker: Pilot study 2 (flexible target and ligand competition) .......... 17
Table S7. Shaker: Final protocol .......... 19
Table S8. Shaker: Refinement of the Cluster Representatives .......... 22
Table S9. Detailed description of final Shaker protocol .......... 25
Table S10. Calculation of RMSD .......... 33
Table S11. Blind docking with association MD: A pilot study .......... 35
Table S12. Computational times of Shaker .......... 36
Table S13. Effect of scoring on Wrapper results .......... 37
Table S14. Shaker results after $MD_{BSA}$: 3ptb .......... 39
Table S15. $MD_F$ results: 0 ns and 20 ns comparison (3ptb) .......... 40
Table S16. $MD_F$ results: 0 ns and 20 ns comparison (1qcf) .......... 42
Table S17. $MD_F$ results: Comparison of ligand conformations at 0 ns and 20 ns (3g5d) .......... 43
Appendix 1 .......... 44
Appendix 2 .......... 47
Appendix 3 .......... 51
Appendix 4 .......... 55
References .......... 59

## Abbreviations

| | |
|---|---|
| **ASA** | Accessible Surface Area |
| **BD** | Blind Docking |
| **CR** | Cluster Representative |
| **$EC_S$** | Exit Criterion of Shaker |
| **$EC_W$** | Exit Criterion of Wrapper |
| **$E_{inter}$** | Intermolecular interaction energy |
| **$E_{LJ}$** | Lennard-Jones potential |
| **$\varepsilon_{ij}$** | AD4 atom parameter: well depth of the Lennard-Jones potential (kcal/mol) |
| **ER** | Elimination Rate |
| **FS** | Filtering Set |
| **$MD_B$** | Molecular Dynamics with backbone position restraints |
| **$MD_{BSA}$** | Molecular Dynamics with backbone position restraints and simulated annealing |
| **$MD_F$** | Molecular Dynamics without restraints (flexible simulation) |
| **N** | Number of ligand copies after Wrapper |
| **$N_F$** | Number of MD simulation frames |
| **$NH_L$** | Number of ligand heavy atoms |
| **PDB** | Protein Data Bank |
| **$pK_a$** | Negative logarithm of dissociation constant ($K_a$) |
| **$R_{ij}$** | AD4 atom parameter: sum of vdW radii of two atoms (Å) |
| **RMSD** | Root Mean Squared Deviation |
| **SA** | Simulated Annealing |
| **solpar** | AD4 atom parameter: atomic solvation parameter |
| **SR** | Shaker Rate |
| **vol** | AD4 atom parameter: atomic solvation volume |

## Table S1. Detailed General Methods

| Methods | | | | | | | | | | |
|---|---|---|---|---|---|---|---|---|---|---|
| **Selection of targets.** Ten systems were used for testing the strategy developed in the present study. Apo, non-complexed structures were used as docking targets for BD calculations, except for System 8, where the holo structure was used. In the case of System 5 another protein tyrosine-protein kinase was used as apo structure similarly to a previous study (Shan, et al., 2011). Ligands from the holo structures were used as references for RMSD calculation. | | | | | | | | | | |
| System # | PDB ID[1] | Res[2] | Target name[3] | PDB ID[3] | Res[4] | AA[5] | RMS[6] | Ligand Name | Ligand B - factor range | MW[7] |
| 1 | 3ptb | 1.70 | bovine β-trypsin | 1s0q | 1.02 | 223 | 0.162 | Benzamidine | 10.6-19.9 | 120 |
| 2a | 3n3l | 2.74 | farnesyl pyrophosphate synthase | 1f7m | 2.3 | 350 | 0.267 | (6-methoxy-1-benzofuran-3-yl) acetic acid (MS0 site 1) | 54.2-95.0 | 206 |
| 2b | 3n3l | 2.74 | farnesyl pyrophosphate synthase | 1f7m | 2.3 | 350 | 0.267 | (6-methoxy-1-benzofuran-3-yl) acetic acid (MS0 site 2) | 91.6-108.3 | 206 |
| 3a | 3hvc | 2.10 | mitogen-activated protein kinase | 1wfc | 2.3 | 366 | 0.652 | 4-[3-(4-fluorophenyl)-1h-pyrazol-4-yl]pyridine (GG5) | 23.5-30.7 | 239 |
| 3b | 4f9w | 2.00 | mitogen-activated protein kinase | 1wfc | 2.3 | 366 | 0.754 | 4-[3-(4-fluorophenyl)-1h-pyrazol-4-yl]pyridine (GG5) | 48.5-85.9 | 239 |
| 4 | 3cpa | 2.00 | carboxy-peptidase | 1m4l | 1.25 | 307 | 0.161 | GY | 10.00 | 256 |
| 5[8] | 1qcf | 2.00 | tyrosine-protein kinase | 1y57 | 1.91 | 452 | 1.027 | 1-ter-butyl-3-p-tolyl-1h-pyrazolo[3,4- | 15.0-27.6 | 281 |

| | | | | | | | | | | |
|---|---|---|---|---|---|---|---|---|---|---|
| | | | Src | | | | | d]pyrimidin-4-ylamine (PP1) | | |
| 6 | 1h61 | 1.40 | pentaerythritol tetranitrate reductase | 1h63 | 1.62 | 364 | 0.107 | Prednisone | 9.8-21.0 | 358 |
| 7 | 2bal | 2.10 | mitogen-activated protein kinase | 1wfc | 2.3 | 366 | 0.602 | [5-amino-1-(4-Fluorophenyl)-1H-Pyrazol-4- yl] [3-(piperidin-4-yloxy) phenyl]methanone (PQA) | 20.7 -37.8 | 380 |
| 8 | 1hvy | 1.90 | thymidylate synthase | | | | | Ralitrexed | 15.2-36.8 | 459 |
| 9[8] | 3g5d | 2.20 | tyrosine-protein kinase Src | 1y57 | 1.91 | 452 | 0.277 | Dasatinib | 25.7-48.2 | 488 |
| 10 | 1be9 | 1.82 | PDZ-domain | 1bfe | 2.3 | 119 | 0.401 | KQTSV | 9.8-35.6 | 544 |

**Notes** [1]Holo system PDB ID; [2]Holo structure resolution; [3]Apo target and PDB ID; [4]Holo structure resolution; [5]Number of amino acid residues in the target [6]RMSD calculated between Cα atoms of the holo and apo structures with pymol structure alignment; [7]Molecular weight; [8]Only cSRC domain was used (259-533 residues).

**Preparation of target molecules for docking**. Missing amino acids from the target structure (in case of System 3), were inserted with Swiss-PdbViewer (Guex and Peitsch, 1997). In cases of missing terminal and non-terminal (1wfc, 1y57) amino acids, acetyl and amide capping groups were added with the Schrödinger Maestro program package v. 9.6 (Release, 2013) to the N- and C-terminus, respectively. In cases of homodimer structures, chain A was used for calculations (1hvy, 1ao6). Target molecules were minimized, as per description in Section "MD Minimization of targets". After the minimization steps, target molecules were prepared for docking, applying a united atom representation for non-polar moieties.

**Preparation of ligands for docking.** The pKa values of the ligand molecules were calculated using the pKa plug-in in Marvin Sketch, v 6.3.0 (ChemAxon). Hydrogens were added according to the correct protonation state at pH 7. Energy minimization was done on hydrogenated

structures using the semi-empirical quantum chemistry program package, MOPAC (MOPAC, 2012), performing a geometry optimization with a 0.001 gradient (kcalmol$^{-1}$Å$^{-1}$) and force calculations with PM3 parameterization. In all cases, the force constant matrices were positive definite. The minimized ligand molecules were prepared for docking, similarly to the target. A united atom representation for non-polar moieties was applied.

**Grid box and docking parameters** Grid box was automatically centered on the target, and grid maps of 200 x 200 x 200 grid points with 0.375 Å spacing were generated. The AutoDock 4.2.3(Morris, et al., 2009) program package was used with Lamarckian Genetic Algorithm (LGA), Gasteiger-Marsili partial charges were added for both, the minimized ligand and target atoms as well. Docking parameters were used as described in the previous study(Hetenyi and van der Spoel, 2011) and input preparations were done as described above.

**Parameters of non-standard residues.** Parameterization of non-amino acid ligands, (benzamidin, MS0, GG5, PP1, Prednisone, PQA, Ralitraxed, Dasatinib), cofactors (FMN, UMP) and non-standard aminoacids in System 8 were necessary because AMBER99SB-ILDN force field does not include molecular mechanics parameters of the ligands used in our study. Charge calculation was performed on the R.E.D Server (Vanquelef, et al., 2011) for the optimized structure, with RESP-A1 charge fitting compatible with AMBER99SB-ILDN force fields. The calculations were performed with the Gaussian09 software (Frisch, et al., 2009), using HF/6-31G* split valence basis set (Krishnan, et al., 1980).

**MD minimization of targets.** Target molecules were minimized using a two-step protocol by the GROMACS 5.0.2 (Abraham, et al., 2015) software package. A steepest descent and conjugated gradient were performed. Using the supplemented targets, we have performed a two-step energy minimization. Simulations were done in AMBER99SB-ILDN force field (Lindorff-Larsen, et al., 2010) with TIP3P explicit water model (Jorgensen, et al., 1983). The target structure was placed in the centre of a cubic box. Distance between the box and the solute atoms was set to 10 Å. The simulation box was filled with water molecules and counter-ions in order to neutralize the total charge of the system. Particle mesh Ewald method was used for long-range electrostatics. The van der Waals and Coulomb cut-offs were set to 11 Å. Convergence threshold of the first step (steepest descent) was set to $10^3$ kJ mol$^{-1}$ nm$^{-2}$, in the second step (conjugant gradient) minimization it was set to 10 kJ mol$^{-1}$ nm$^{-2}$. Final structures obtained from the energy minimization can be launched into further MD calculations (for Shaker), or into docking calculations (for Wrapper).

**General parameters for production trajectories (MD)**

MD simulations of the ligand-target complexes were performed using the GROMACS program package with force field settings described at Energy Minimization. All frames generated in MD trajectory were extracted. Position restraints were applied on the backbone heavy atoms (except $MD_F$) with a force constant of $10^3$ kJmol$^{-1}$nm$^{-2}$ during the whole MD simulation. In case of System 4, distance restraints between the ion (coordinated structural $Zn^{2+}$) and the

coordinating residues was needed to maintain proper geometry. PME (Particle Mesh-Ewald) summation was used for long-range electrostatics. Van der Waals and Coulomb interactions had a cut-off at 11 Å. For temperature coupling the velocity rescale algorithm was used. Solute and solvent were coupled separately with a reference temperature of 300 K (except in case of $MD_{BSA}$) and a coupling time constant of 0.1 ps. Pressure was coupled the Parrinello-Rahman algorithm with a coupling time constant of 0.5 ps, compressibility of $4.5\times10^{-5}$ $bar^{-1}$ and reference pressure of 1 bar. Structures were exported at every 0.1 ns time step.

**Specific parameters for production trajectories (MD)**

In addition to the General parameters of MD, specific parameters regarding the duration, position restraint, and temperature scheme will be described for each MD type in the Shaker protocol.

**Shaker**

**$MD_B$** A 5-ns-long MD simulation was performed, using backbone heavy atom position restraints, with a $10^3$ $kJmol^{-1}nm^{-2}$ force constant. Export of coordinates at every 0.1 ns time step, resulted in a total of 51 frames. All other simulation parameters, were unchanged and used as presented in Section General parameters for production trajectories (MD).

**$MD_{BSA}$.** A 20-ns-long simulated annealing MD was performed, using backbone heavy atom position restraints, with a $10^3$ $kJmol^{-1}nm^{-2}$ force constant. Export of coordinates at every 0.1 ns time step, resulted in a total of 201 frames. Simulated annealing temperature was rescaled and controlled in the same way for each temperature group in GROMACS (both solvent and solute). Based on the differences at the molecular weight (MW) level of the ligands, SA was performed using two temperature increase schemes up to 50°C (Scheme 1, ligand MW ≤ 300) or 80°C (Scheme 2, ligand MW ≥300).

**Scheme 1**

| Time (ps) | 0 | 2500 | 5000 | 10000 | 15000 | 17500 | 20000 |
|---|---|---|---|---|---|---|---|
| T (K) | 300 | 310.15 | 323.15 | 323.15 | 323.15 | 310.15 | 300 |

**Scheme 2**

| Time (ps) | 0 | 1250 | 2500 | 3750 | 5000 | 10000 | 15000 | 16250 | 17500 | 18250 | 20000 |
|---|---|---|---|---|---|---|---|---|---|---|---|
| T (K) | 300 | 315.15 | 330.15 | 343.15 | 353.15 | 353.15 | 353.15 | 343.15 | 330.15 | 315.15 | 300 |

All other simulation parameters, were unchanged and used as presented in Section General parameters for production trajectories (MD).

**$MD_F$.** A 20-ns-long MD simulation was performed, without any position restraints on the target. Export of coordinates at every 0.1 ns time step, resulted in 201 total frames. All other simulation parameters, were unchanged and used as presented in Section General parameters for production trajectories (MD).

## Table S2. Wrapper: A pilot study

| Goals |
|---|
| Development of an algorithm which performs several fast blind docking (BD) cycles using AutoDock 4.2.3 and systematically covers (wraps) the entire surface of the target with a monolayer of ligand copies. Notably, current randomized search algorithms cannot guarantee systematic exploration of the entire target surface for the binding pockets by default. In the ligand monolayer the ligands should exclusively interact with the target, minimizing the ligand-ligand interactions. |
| **Method** |
| To achieve the above goals, two trials were performed in this pilot study.<br>**Trial 1.** Multiple and consecutive BD cycles were performed using the original settings and force field parameters as described in (Hetenyi and van der Spoel, 2006). Each BD cycle results in 100 docked ligand copies which are ordered by energy structurally clustered (Hetenyi and van der Spoel, 2006) and a final set of ligand copies is retained for the next cycle. After each docking cycle the obtained m Cluster Representatives (CRs) were merged with the target and the target-ligand$_m$ complex was used as the receptor input for the next docking cycle. This cyclic process continued until one of the exit criterion ($EC_W$-Exit criterion of Wrapper) is reached:<br>- uncovered protein surface below one percent of its total (ligand-free, initial) surface area or;<br>- positive target-ligand interaction energy in every cluster representative (Fig. 1 in main text).<br>**Trial 2.** A new atom type (X) was assigned to all target amino acid atoms that were situated within a 3.5 Å radius distance from the docked CR atoms. AutoDock 4.2 force field parameters of atom type X ($R_{ij}$, $\varepsilon_{ij}$, vol, solpar) were set to zero with the purpose to avoid any type of interaction between the marked target surface and the following cycle CRs. The Coulomb term was also switched off by setting the partial charges of atoms X to zero. Since atom parameters were set to zero, no interaction was possible between ligand and atoms with type X on the target surface. |
| **Results** |
| **Trial 1.** After multiple docking cycles, the ligands started to self-aggregate, and a monolayer ligand copies on the target surface was not possible to obtain (see figure below). We concluded that a repulsive potential would be necessary to avoid such unwanted ligand-ligand interactions along with an attractive interaction between the ligands and the target at the same time. |

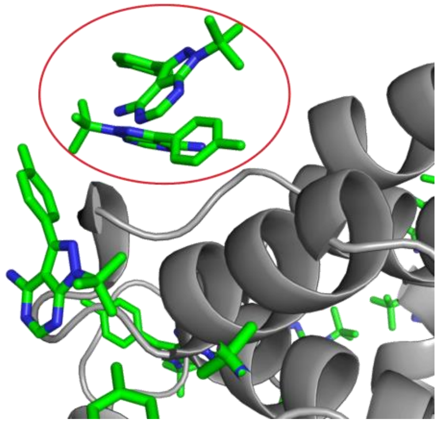

**Self-aggregated ligand docking.**

Protein tyrosine kinase (System 5) is represented with grey cartoon, PP1 with green sticks. After two docking cycles, aggregates can be observed between ligands, although free (unbound) surface is still available on the target.

**Trial 2.** After multiple docking cycles, using the new atom type, the result was rather an artifact, because the ligands started to interact with the non-modified aminoacids from the interior of the target, obtaining unrealistic clashes (see figure below). Thus, simple switch (zeroing) of all interactions did not help.

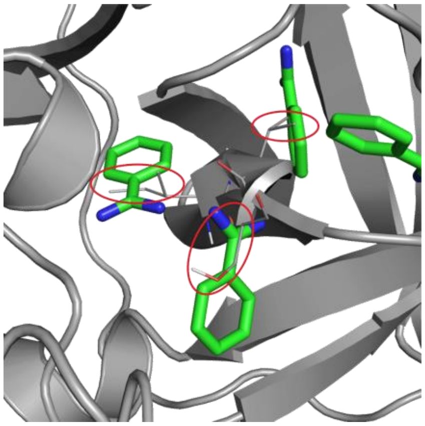

**Artefact target-ligand clashes.** The target beta trypsin (System 1 in Table 1) is represented with grey cartoon, inhibitor benzamidine is in green sticks. After two docking cycles, clashes between the target residues and benzamidine can be observed in three spots with red circles.

**Conclusions**

The above trials were not able to wrap the target in a ligand monolayer, as reducing ligand-ligand interactions, and maximizing ligand-target interactions was not successful. For this reason, we continued to improve our method and reached a final solution described in Table S3.

## Table S3. Wrapper: Development of force field parameters

| Goals |
|---|
| Previous attempts (Table S2) showed that building of a ligand monolayer requires blockage of formation of overlaps (aggregates) between the docked ligand copies (Trial 1, Table S2) and maximization of new target-ligand interactions. At the same time, clashes observed in Trial 2 (Table S2) should be also avoided. To accomplish these requirements in the same wrapper cycle, further experimenting on the modification of the docking force field was necessary. |
| **Results** |
| We decided to switch off only the electrostatic terms by zeroing the partial charges of the new target atom type (X, Table S2) to be excluded from successive docking cycles. Furthermore, the new atom type (X) was added to the cluster representative ligand copies, obtained from docking cycles. At the same time, a systematic calibration of the Lennard-Jones (LJ) parameters of X was performed to achieve the above goals.<br>The LJ parameters of atom X were calibrated considering the pairwise interaction potential ($V_{XY}$) with three common atom types (Y=O, C and H). A systematic search of both $\varepsilon_X$ and $R_X$ was conducted on a physically meaningful interval of LJ parameters (see below). Numerous docking runs were performed to check the physical effect of the selected LJ parameters. A pre-defined value of r = 2 Å was used as a minimal distance where short range repulsive effects should act.<br>$V_{XY} = \varepsilon\left[\left(\frac{R_{XY}}{r}\right)^{12} - 2\left(\frac{R_{XY}}{r}\right)^{6}\right]$<br>where<br>$R_{XY} = \frac{R_X + R_Y}{2}$<br>$\varepsilon_{XY} = \sqrt{\varepsilon_X \varepsilon_Y}$<br>where,<br>$\varepsilon_{XY}$ = potential well depth at equilibrium between particles of types X and Y<br>$R_{XY}$= inter-nuclear distance at equilibrium between particles of types X and Y<br>r= actual distance between the two atoms |

**LJ Parameters**

| Atom type | R Å | ε kcal/mol |
|---|---|---|
| Y = O[1] | 3.20 | 0.20 |
| Y = C[1] | 4.00 | 0.15 |
| Y = H[1] | 2.00 | 0.20 |
| X[2] | 2.00-5.00 | $10^{-5}$-$10^{-1}$ |

**Notes**

[1] For C, H and O default AD4 parameters were used (Morris, et al., 2009)

[2] For X, the search interval was performed with 0.1 precision step.

**Selection of $\varepsilon_X$**

Based on the above (known) atomic LJ parameters of atom types Y, and the pre-defined r = 2 Å, $V_{XO}$ was calculated for three $\varepsilon_X$ values (below) and stepping $R_x$ by 0.1 Å in the above search interval. $\varepsilon_X$ values between $10^{-5}$-$10^{-3}$ kcal/mol were considered as a desired maximal repulsion potential value of $V_{XO}$≈1 kcal/mol could be obtained in this interval at physically relevant $R_X$ values. The docking trials showed that potential value of 1 kcal/mol was enough to reach the repulsion at atoms of type X during docking, and therefore, setting of maximal $V_{XO}$ larger than 1 kcal/mol would not be useful to accomplish the above Goals.

Finally, the following three scenarios differing by one order of magnitude in $\varepsilon_X$ were investigated so as to reach the $V_{XO}$≈1 kcal/mol (Fig. 2 in the main text).

1) Large $R_X$, small $\varepsilon_X$ (red)

2) Medium Rx, medium $\varepsilon_X$ (green)

3) Small Rx, large $\varepsilon_X$(blue)

Scenario 2 was identified including an optimal magnitude of $\varepsilon_X=10^{-4}$ kcal/mol with a maximal $R_X$ between 3.0 and 3.5 Å (approximately distance limit between heavy atoms in a H-bond). In this case, available target surface is optimally used without generating large ligand-free zones in the monolayer. If maximal $R_x$ was too large (Scenario 1) then the repulsion zone around the docked ligand copies would become too large resulting in large ligand-free zones, i.e. a non-optimal arrangement of the ligand copies in the monolayer. On the other hand, if maximal $R_X$ was too small (Scenario 3), then unwanted effects such as aggregation between docked ligand copies would happen similar to Trial 1 in Table S2.

**Calculated $V_{XO}$ (kcal/mol)**

| $\varepsilon_X$ / $R_X$ | **Large $R_X$, small $\varepsilon_X$** | **Medium Rx, medium $\varepsilon_X$** | **Small Rx, large $\varepsilon_X$** |
|---|---|---|---|
| | **$10^{-5}$** | **$10^{-4}$** | **$10^{-3}$** |
| 2 | **0.02** | **0.06** | **0.19** |
| 2.1 | **0.03** | **0.08** | **0.26** |
| 2.2 | **0.03** | **0.11** | **0.35** |
| 2.3 | **0.05** | **0.14** | **0.45** |
| 2.4 | **0.06** | **0.19** | **0.59** |
| 2.5 | **0.08** | **0.24** | **0.75** |
| 2.6 | **0.10** | **0.30** | **0.96** |
| **2.7** | **0.12** | **0.38** | **1.21** |
| 2.8 | **0.15** | **0.48** | 1.51 |
| 2.9 | **0.19** | **0.60** | 1.88 |
| 3 | **0.23** | **0.74** | 2.33 |
| 3.1 | **0.29** | **0.91** | 2.86 |
| **3.2** | **0.35** | **1.11** | 3.51 |
| 3.3 | **0.43** | 1.35 | 4.27 |

| | | | |
|---|---|---|---|
| 3.4 | **0.52** | 1.64 | 5.19 |
| 3.5 | **0.63** | 1.98 | 6.27 |
| 3.6 | **0.76** | 2.39 | 7.56 |
| 3.7 | **0.91** | 2.87 | 9.07 |
| **3.8** | **1.09** | 3.43 | 10.85 |
| 3.9 | 1.29 | 4.09 | 12.95 |
| 4 | 1.54 | 4.87 | 15.40 |
| 4.1 | 1.83 | 5.77 | 18.26 |
| 4.2 | 2.16 | 6.83 | 21.59 |
| 4.3 | 2.55 | 8.06 | 25.47 |
| 4.4 | 3.00 | 9.48 | 29.97 |
| 4.5 | 3.52 | 11.12 | 35.18 |
| 4.6 | 4.12 | 13.03 | 41.19 |
| 4.7 | 4.81 | 15.22 | 48.13 |
| 4.8 | 5.61 | 17.75 | 56.12 |
| 4.9 | 6.53 | 20.65 | 65.29 |
| 5 | 7.58 | 23.97 | 75.81 |

**Selection of $R_X$**

The above calculation of $V_{XO}$ was repeated for atom types Y= C and H, at the optimal $\varepsilon_X=10^{-4}$ kcal/mol and the results are shown in Appendix 1. Thus, the resulted maximal $R_X$ values were 3.2 Å, 2.5 and, 5.0 of which an average of 3.6 Å was calculated and implemented in the AutoDock 4.2 force filed as a final $R_X$ allowing an optimal repulsion zone around excluded (covered target and docked ligand) atoms.

## Conclusions

The calibrated Lennard-Jones parameters result in a repulsive potential allowing an optimal coverage of a target by a ligand monolayer.

## Table S4. Filters of the Shaker algorithm

| **Methods** | |
|---|---|
| Shaker algorithm uses the distance-based ($d_C$) and interaction energy-based ($E_{inter}$) **basic filters** calculated directly from the frames of simulation trajectories. A trajectory contains $N_F$ frames, where the actual value of $N_F$ can be tuned. The value of NF used in the present study is specified for each MD simulation type in Table S1. A frame includes all structural (coordinate, atom type, etc.) information of the target-ligand complex. Further, **derived filters** were calculated from the basic filters. Finally, the filters were combined into **Filter set** and used in pilot studies and in the final Shaker algorithm. Note that serial number of a filter (Filter #) indicate the priority of a filter in the filtering sequence of the filter set as described below. | |
| **Basic filter** | **Definition** |
| **$d_{C,f}$** | The smallest interatomic distance measured between the heavy atoms of the target and ligand molecules.<br>f=1,2,3,…,$N_F$ where $N_F$ is the number of frames in the MD trajectory |
| **$E(LJ)_{inter,\,f}$** | Lennard-Jones potential calculations were carried out using Amber (Wang, et al., 2012) van der Waals parameters.<br>$E(LJ)_{inter,f} = \sum_{i,j}^{N_T N_L} \left( \frac{A_{ij}}{r_{ij}^{12}} - \frac{B_{ij}}{r_{ij}^{6}} \right)$<br>where<br>$A_{ij} = \varepsilon_{ij} R_{ij}^{12}$<br>$B_{ij} = 2\varepsilon_{ij} R_{ij}^{6}$<br>where,<br>$R_{ij} = R_i + R_j$<br>$\varepsilon_{ij} = \sqrt{\varepsilon_i \varepsilon_j}$<br>$N_T$: number of target atoms<br>$N_L$ : number of ligand atoms<br>$R_i$, $R_j$, $\varepsilon_i$, $\varepsilon_j$: parameters specific for the interacting for the i[th] ligand and the j[th] target atom pairs in frame f<br>f=1,2,3,…,$N_F$ where $N_F$ is the number of frames in the MD trajectory |
| **Derived filter** | **Definition** |
| **$d_{c,AVG}$** | $d_{C,AVG} = \frac{1}{N_F} \sum_{i=1}^{N_F} d_{C,f}$<br>f=1,2,3,…,$N_F$ where $N_F$ is the number of frames in the MD trajectory |
| **$E(LJ)_{inter,\,AVG}$** | $E(LJ)_{inter,AVG} = \frac{1}{N_F} \sum_{i=0}^{N_F} E(LJ)_{inter}$ |

| Filter set | Filter # | Filter & Threshold | Description | Used in |
|---|---|---|---|---|

| | | | | |
|---|---|---|---|---|
| **FS** | 1 | $d_{C,AVG}$ < 3.50 Å | $d_C$ and E(LJ) filters were calculated for all $N_F$ trajectories and time steps, and used in four consecutive filtering steps. The distance metrics ($d_C$) were used in two consecutive filtering steps. With distance Filter #1, it could be ensured that those ligands that during the trajectory are mainly away from the target surface, or binding weakly ($d_{C,AVG}$ values) are filter out initially. Ligands that are not on the target surface at the final frame of the simulation ($N_F^{th}$ values) will be filtered out in the Filter #2 ($d_{C,NF}$). The purpose of filter #3 and #4 was, to eliminate the weak binders, and those positions, where the binding energy increased more than 25% (from the initial frame) during the simulations. Such increase suggests an unstable binding pose and site. | Shaker pilot study and final protocol: Table S7, S8 and S9. |
| | 2 | $d_{C,NF}$ < 3.50 Å | | |
| | 3 | $E(LJ)_{inter,\ AVG}$ < 25 % from first frame | | |
| | 4 | $E(LJ)_{inter,\ NF}$ < 25 % from first frame | | |

## Table S5. Shaker: Pilot study 1: $MD_B$

| **Goals** |
|---|
| The purpose of the Shaker protocol is to eliminate the excess of ligands, keep the functional binding sites highly ranked, while also ensuring stable MD simulations. In this strategy, a series of MD simulations are performed for the target-ligand$_N$ complex. That is, the target wrapped in N ligand copies is placed in a box filled with water and subjected to several short MD runs. |
| **Methods** |
| Multiple $MD_B$ cycles were performed to eliminate the ligand excess. Each $MD_B$ cycle, was followed by filtering with the FS (Table S4). This was necessary to eliminate those ligands that have dissociated from the target surface during the simulations.<br>The Elimination Rate (ER) is defined as the ratio of the number of ligands eliminated in Shaker (N-n) and the total number of ligands after wrapper (N), in order to have a measure of shaker efficiency and it is used as system-independent threshold in the Shaker. The calculated ER was used to set up the Shaker exit criterion ($EC_S$). ER is naturally connected to SR as it follows.<br>$ER=1-n/N=1-SR^{-1}$<br>Shaker protocol started with 5-ns-long backbone restrained $MD_B$, to grossly shake off the weakly bound ligand excess. If this initial $MD_B$ was not enough to reach ER $EC_S$ #1, $MD_B$ cycles were continued with multiple 10-ns simulations, until ER $EC_S$ #2 was achieved.<br>After each MD filtering, ER was calculated, and MD simulation cycles were discontinued, once the final $EC_S$ #2 was reached<br>The filtering criteria were used as presented at FS (Table S4), and the MD simulation conditions are detailed in Table S1.<br>Our protocol was launched on two test systems (3hvc and 1qcf). For these systems, we have investigated the number of cycles that would be necessary in order to achieve the targeted final ER value ($EC_S$ #2 = 0.90). It was considered that by eliminating one-ninth from the initial ligand numbers, will eliminate ligand access while, still keeping all the ligand conformations that bound to potential functional binding sites. |

| ER $EC_S$ # | ER | $MD_B$ Simulation Time (ns) | Restraint on the target |
|---|---|---|---|
| 1 | 0.25 | 5 | Backbone position restraint |
| 2 | 0.90 | 10 | Backbone position restraint |

| **Results** |
|---|
| In the table below, it can be observed, that a high number of Shaker cycles (5 and 6 respectively) were necessary on the test systems, without achieving the desired final ER ($EC_S$ #2=0.90), and therefore, further $MD_B$ cycles were not started even if the $EC_S$ #2 was not achieved. |

| PDB ID | Cycles necessary to achieve ER $EC_S$ #2 | Final ER achieved |
|---|---|---|

| | | |
|---|---|---|
| 3hvc | 5 | 0.77 |
| 1qcf | 6 | 0.87 |
| **Conclusions** | | |
| Due to the high number of cycles necessary to achieve $EC_S$ #2, in the next approach we tried full flexibility on the target (see Table S6). | | |

## Table S6. Shaker: Pilot study 2 (flexible target and ligand competition)

**Goals**

The approach and goals remained the same as stated in Table S5, however the purpose in this pilot study, was to increase the efficiency of our strategy, compared to the previous pilot study. Compared to Table S5, changes were made in the $EC_S$ and MD simulation conditions, by introducing target flexibility, in the detriment of backbone fixation of the target.

**Methods**

$EC_S$ #1, and simulation condition in the first round of MD, is the same as in Table S5. After the first $EC_S$ was met, 10 ns long $MD_B$ simulations were performed, until the 0.75 of the ligands were eliminated ($EC_S$ #2) from the starting target-ligand$_N$ complex. For the third stage of MD, total flexibility was set on the target ($MD_F$), until 0.90 of the ligands were eliminated ($EC_S$ #3). This protocol was implemented with the purpose of conformational refinement, and for speeding up desorption of the ligands, trapped in local energy minima due to receptor partial rigidity.

| $EC_S$ # | ER | Simulation Time (ns) | Restraint on the target |
|---|---|---|---|
| 1 | 0.25 | 5 | Backbone position restraint |
| 2 | 0.75 | 10 | Backbone position restraint |
| 3 | 0.90 | 10 | No restraint |

**Results**

In the proximity of the binding sites, we have observed, that ligands are competing between each other for the binding site, therefore leaving a full flexibility on the target, caused structural deformations. These structural deformations on the target, are artefacts that are in the detriment of further validations, and comparison possibilities with the reference, crystallographic structures. During $MD_F$ simulations, ligand self-aggregates were observed on the target surface, which due to total target flexibility, made the system prone to target deformation (see Figure below).

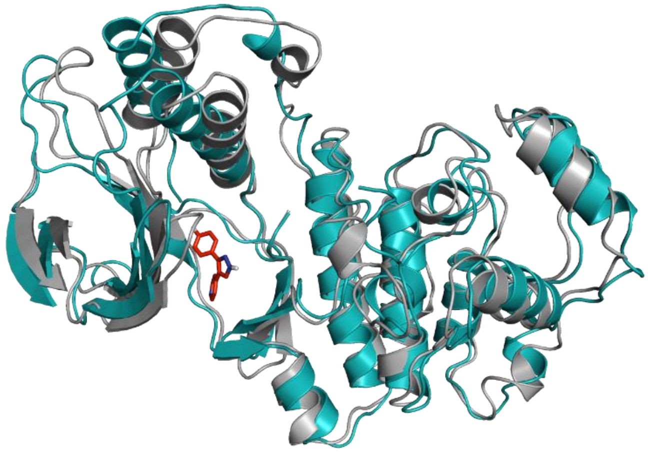

| **System 3 (3hvc) after 8 cycles of MD simulations.**<br>Reference ligand position is marked with red sticks, target conformation before MD simulations is represented with grey cartoon, and target conformation after the flexible simulations, is represented with teal cartoon. It can be observed, that target conformation around the ligand is altered. A 1.9 Å RMSD was obtained after a Cα alignment of the structures. | | |
|---|---|---|
| **PDB ID** | **Cycles** | **Final ER achieved** |
| 3hvc | 8 | 0.88 |
| 1qcf | 4 | 0.90 |
| **Conclusion** | | |
| Although the targeted ER value (0.90) was obtained in case of 1qcf, due to the fact that ligand competition and structural deformations were observed, we have further improved the MD conditions in our final Shaker strategy (see Table S7). | | |

## Table S7. Shaker: Final protocol

| **Goals** | | | |
|---|---|---|---|
| As the pilot experiments (Table S5 and S6) described above, were not satisfactory in terms of Shaker efficiency, simulated annealing MD simulations ($MD_{BSA}$) were introduced in our final Shaker protocol. Backbone restraint was kept to avoid structural deformations (Table S6). The main purpose of $MD_{BSA}$, was to increase the ER and speed up the desorption process. | | | |
| **Methods** | | | |
| Based on the differences at the molecular weight (MW) level of the ligands, SA was done, using two temperature increase strategies, up to 50°C (ligand MW ≤ 300) or 80°C (ligand MW ≥ 300). After the ligand excess was eliminated in multiple $MD_{BSA}$ cycles, a clustering step was performed, using the last frames of the surviving ligands.<br>In the table below, we can observe the simulation conditions and the $EC_S$, set for the final Shaker protocol. Similarly to the pilot studies (Table S5 and Table S6), the $EC_S$ #1 and the simulation conditions remained unchanged for the initial rough shake. The $EC_S$ #2 was set to 0.75 in our validation. After the $EC_S$ #2 was achieved, a clustering step was included.<br>Prior to the clustering step, target-ligand $E(LJ)_{inter}$ interaction (Table S4) was calculated for the entire simulation. Using these calculated $E(LJ)_{inter}$ interactions, the ligand structures from the last frames of the surviving trajectories are clustered and ranked in a descending order of interaction energy. The ligands with the lowest $E(LJ)_{inter}$ are selected as cluster representatives among the cluster members. A minimum 7 Å cut-off distance between the heavy atoms of the cluster representatives was set, in order to avoid clashes between the CRs. | | | |
| **$EC_S$ #** | **ER** | **Time (ns)** | **Restraint on the target** |
| 1 | 0.25 | 5 | Backbone position restraint |
| 2 | 0.75 | 20 | Backbone position restraint |
| | | | |
| **Results** | | | |
| In the table below, we can observe, that markedly fewer Shaker cycles were necessary to obtain the same or even better ER, with this final Shaker strategy, compared to the previous two pilot experiments (see Table S5 and S6). | | | |
| **PDB ID** | **Cycles necessary to achieve the $EC_S$ #2** | **ER achieved after $MD_{BSA}$** | **ER achieved after clustering** |
| 3hvc | 2 | 0.79 | 0.91 |
| 1qcf | 3 | 0.83 | 0.92 |
| **ER – on all Test Systems**<br>The below table contains the ER results obtained after $MD_{BSA}$, and after the clustering step. In the Main text, Table 2 contains the SR values that were obtained after the clustering step. | | | |

| System # | PDB ID | CLS[1] | n[2] | ER after $MD_{BSA}$[3] | ER after Clustering[3] |
|---|---|---|---|---|---|
| 1a | 3PTB | 6 | 6 | 0.91 (after $MD_B$) | 0.91 |
| 1b[4] | 3PTB | 5 | 4 | 0.93 (after $MD_B$) | 0.95 |
| 1c[4] | 3PTB | 6 | 5 | 0.92 (after $MD_B$) | 0.93 |
| 2 | 3N3L | 18 | 13 | 0.94 | 0.96 |
| 3a | 3HVC | 46 | 21 | 0.79 | 0.91 |
| 3b | 3HVC | 46 | 21 | 0.79 | 0.91 |
| 4[5] | 3CPA | 12 | 8 | 0.92 | 0.95 |
| 5 | 1QCF | 25 | 12 | 0.83 | 0.92 |
| 6[6] | 1H61 | 26 | 12 | 0.78 | 0.90 |
| 7 | 2BAL | 26 | 12 | 0.79 | 0.90 |
| 8 | 1HVY | 25 | 10 | 0.76 | 0.91 |
| 9[7] | 3G5D | 23 | 10 | 0.75 | 0.89 |
| 10 | 1BE9 | 11 | 4 | 0.77 | 0.92 |

[1]Total ligands remained after Shaker with $MD_{BSA}$

[2]Total ligands remained after Clustering

[3] ER was calculated in two stages.

[4]1b and 1c are referring to 3PTB when docking was performed with different seed for data reproduction purposes.

[5]Final clustering was done using van der Waals and Coulomb interactions due to $Zn^{2+}$ interactions with the ligand.

[6]Docking was performed, after rescoring of the 100 conformations with, $E_{LJ}$ potential instead of AutoDock 4.2 scoring function

[7]Final clustering was done with 6 Å distance limit between clusters

## RMSD – on all Test Systems

The below table contains the RMSD results, after the $MD_B$, and after refinement with $MD_F$. It can be observed that $MD_F$ usually improved the RMSD values. In some cases, serious improvement can be observed from 6.84 to 1.69 (System 10), but slight improvement can be observed in most of the cases.

| System Nr | PDB ID | After $MD_{BSA}$ | | After $MD_F$ | |
|---|---|---|---|---|---|
| | | $RMSD_{CR}$ [1] | $RMSD_{MD}$ [1] | $RMSD_{CR}$ [2] | $RMSD_{MD}$ [2] |
| 1a | 3PTB | 0.66 | 0.79 | 1.27 | 1.60 |
| 1b | 3PTB | 0.86 | 0.36 | - | - |
| 1c | 3PTB | 0.92 | 0.50 | - | - |
| 2 | 3N3L | 3.41 | 3.75 | 3.13 | 3.17 |
| 3a | 3HVC | 4.44 | 4.14 | 5.54 | 5.00 |
| 3b | 3HVC | 4.54 | 4.93 | 4.66 | 4.08 |
| 4 | 3CPA | 3.86 | 4.80 | 3.79 | 4.45 |
| 4[3] | | | | 3.29 | 2.70 |
| 5 | 1QCF | 1.00 | 1.05 | 1.43 | 1.32 |
| 6 | 1H61 | 1.22 | 2.33 | 0.66 | 2.17 |
| 7 | 2BAL | 3.01 | 2.96 | 3.09 | 2.70 |

| | | | | | |
|---|---|---|---|---|---|
| 8 | 1HVY | 2.95 | 3.77 | 2.33 | 2.57 |
| 9 | 3G5D | 2.34 | 1.93 | 1.85 | 1.66 |
| 10 | 1BE9 | 6.84 | 6.25 | 1.69 | 1.96 |

**Notes**

[1] RMSD ($C_\alpha$ alignment, using aminoacids listed in Table S10) values obtained after $MD_{BSA}$, before the $MD_F$ step.

[2] RMSD ($C_\alpha$ alignment, using aminoacids listed in Table S10) values obtained after $MD_F$, refinement step.

[3] $MD_F$ was continued until 100 ns in this case

## Conclusions

Multiple approaches were studied and tested (See Table S5 and S6), before finding the most effective protocol. In our final protocol, we have included the simulated annealing, in order to increase the dissociation speed, but maintained a backbone restraint during the simulated annealing, to avoid any structural deformation of the target. The implemented final protocol was the most effective from the ER perspective, and also provided highly ranked conformations, with good RMSD. As part of the Shaker, after performing the clustering step an additional refinement step with $MD_F$ (MD simulations, with full protein flexibility), was performed for each cluster representative. This additional step was carried out, in order to allow the necessary conformational changes and induced fit movements for the final ligand binding to take place.

## Table S8. Shaker: Refinement of the Cluster Representatives

**Goal**

In the final Shaker protocol, $MD_{BSA}$ was performed with backbone restraint, to limit target flexibility, but additional $MD_F$ simulation was included in the Shaker protocol, to refine the conformation of the cluster representatives. No position restraint was set on the target, in $MD_F$ step to allow any induced-fit movements to take effect. Since this step is considered a refinement step, the duration of the simulations at this stage can be entirely customizable, depending on the user's need, and computational availability.

**Methods**

The target - cluster representative complexes were subjected to flexible 20-ns long MD simulations (exception in case of System 4), for ligand conformation refinement. After the simulations, we have evaluated the RMSD (see Table S10 for RMSD calculation methods) and the target – ligand interaction energy changes from the end of the simulation. Based on the calculated target-ligand interaction energy ($E_{LJ}$) at the final time step (frame) of the simulation, a re-ranking was performed.

**Results**

**RMSD fluctuation**

RMSD was calculated for the cluster representatives, that after the $MD_{BSA}$, were the closest to the reference ligand (Table 2 – Column: #Rank). During the 20–ns long refinement simulations, RMSD was calculated at each time step (frame). Two types of RMSDs were calculated, using the two types of reference structures ($RMSD_{CR}$ and $RMSD_{MD}$–see Table S10).

In the table below, RMSD values were collected for the final frame of $MD_F$, the lowest (Min), highest (Max), average, and the standard deviation of the RMSD during the simulations.

The difference between the minimum and maximum RMSD in some cases, (System 6: 0.59 - 2.23 or 1.53 - 3.58) can demonstrate that considering only the RMSD calculated at the final frame, from an entire simulation process is not always relevant. This RMSD variation, can be also in close relation with the experimental B-factor value. For example, in case of System 2, the difference between the min and max RMSD varies markedly (2.50-5.05 or 2.41–5.27), which is in good agreement with the B-factor min and max value variations (54.23-94.94, see Table S1).

| System # | PDB ID | $RMSD_{CR}$ (Å) | | | | | $RMSD_{MD}$ (Å) | | | | |
|---|---|---|---|---|---|---|---|---|---|---|---|
| | | Final frame[1] | Min[2] | Max[3] | Avg[4] | Stdv[5] | Final frame[1] | Min[2] | Max[3] | Avg[4] | Stdv[5] |
| 1a | 3PTB | 1.27 | 1.24 | 1.59 | 1.39 | 0.06 | 1.60 | 1.33 | 1.84 | 1.52 | 0.11 |
| 1b | 3PTB | - | - | - | - | - | - | - | - | - | - |
| 1c | 3PTB | - | - | - | - | - | - | - | - | - | - |
| 2 | 3N3L | 3.13 | 2.50 | 5.05 | 3.48 | 0.62 | 3.17 | 2.41 | 5.27 | 3.43 | 0.71 |
| 3a | 3HVC | 5.54 | 5.42 | 7.48 | 6.05 | 0.38 | 5.00 | 4.98 | 6.11 | 5.55 | 0.22 |
| 3b | 3HVC | 4.66 | 4.02 | 7.00 | 4.19 | 0.33 | 4.08 | 3.63 | 5.83 | 4.19 | 0.33 |

| | | | | | | | | | | |
|---|---|---|---|---|---|---|---|---|---|---|
| 4[6] | 3CPA | 3.29 | 2.88 | 5.92 | 3.61 | 0.46 | 2.71 | 2.51 | 6.00 | 3.57 | 0.46 |
| 5 | 1QCF | 1.43 | 1.01 | 1.71 | 1.25 | 0.15 | 1.32 | 0.98 | 1.72 | 1.25 | 0.14 |
| 6 | 1H61 | 0.66 | 0.59 | 2.23 | 1.21 | 0.36 | 2.17 | 1.53 | 3.58 | 2.61 | 0.43 |
| 7 | 2BAL | 3.09 | 2.56 | 5.36 | 3.98 | 0.66 | 2.70 | 2.08 | 5.02 | 3.65 | 0.72 |
| 8 | 1HVY | 2.33 | 2.22 | 3.32 | 2.67 | 0.22 | 2.57 | 2.53 | 4.08 | 3.11 | 0.33 |
| 9 | 3G5D | 1.85 | 0.87 | 2.78 | 2.02 | 0.36 | 1.66 | 0.92 | 2.65 | 1.88 | 0.38 |
| 10 | 1BE9 | 1.69 | 1.26 | 8.11 | 2.36 | 1.39 | 1.96 | 1.09 | 8.01 | 2.45 | 1.39 |

**Notes**

[1] RMSD from the final frame of $MD_F$

[2] The lowest RMSD in the simulation

[3] The highest RMSD in the simulation

[4] The average RMSD during the simulation

[5] The standard deviation during the simulation

[6] The RMSD results are written for the 100 ns log simulation in this case.

## Re-ranking

In the table below, results obtained after MD refinement, can be observed for cluster representative that started the $MD_F$ with the smallest RMSD. After $MD_F$, the total number of cluster representatives is not changed. The Rank# of the ligand with the best RMSD is modified by re-ranking based on the final interaction energy (Appendix 3).

In case of System 10, after $MD_F$, the cluster representative that was initially ranked in the second position, was promoted to the first one, due to considerable improvement in target-ligand interaction energy (from -27.11 kcal/mol to -46.59 kcal/mol). For this system, a drastic RMSD improvement was also observed (Table S7).

Another approach on System 10 was to submit the conformation with the best RMSD obtained from docking, directly into $MD_F$ simulation. The results from this experiment can be observed at system 10b. Comparing the results from 10a with 10b, from energy and RMSD point of view, we can observe, that ligand conformation in 10b had better interaction with the target, and lower RMSD at the beginning of the simulation. Although it was more a favorable initial conformation, the pentapeptide in 10b did not found the reference position by the end of the simulation (RMSD: 11.3 Å).

| system # | PDB ID | Rank # after $MD_{BSA}$ | Rank # after $MD_F$ | $E_{LJ}$ (before minimization) | $E_{LJ}$ (after minimization) | $E_{LJ}$(after $MD_F$) | $RMSD_{CR}$ initial | $RMSD_{CR}$ final |
|---|---|---|---|---|---|---|---|---|
| 1a | 3ptb | 1 | 1 | -18.4 | -14.4 | -16.0 | | |
| 2 | 3n3l | 2 | 4 | -29.0 | -30.2 | -20.8 | | |
| 3a | 3hvc | 3 | 4 | -29.6 | -28.0 | -26.0 | | |
| 3b | 3hvc | 9 | 12 | -17.7 | -15.4 | -17.3 | | |
| 4 | 3cpa | 1 | 1 | -45.6 | -47.1 | -44.6 | | |
| 5 | 1qcf | 2 | 1 | -34.0 | -32.6 | -39.0 | | |
| 6 | 1h61 | 1 | 2 | -41.5 | -34.8 | -34.4 | | |

| | | | | | | | | |
|---|---|---|---|---|---|---|---|---|
| 7 | 2bal | 4 | 4 | -32.0 | -33.9 | -36.9 | | |
| 8 | 1hvy | 1 | 1 | -46.6 | -45.2 | -51.0 | | |
| 9 | 3g5d | 2 | 1 | -41.5 | -43.9 | -48.3 | | |
| 10a | 1be9 | 2 | 1 | -27.1 | -29.7 | -46.6 | | |
| 10b | 1be9 | | | | -33.8 | -27.6 | 6.6 | 11.3 |

**Conclusions**

This refinement step ($MD_F$) is crucial in cases where conformational changes occur on the target upon ligand binding (induced-fit). During Shaker the target is constrained with backbone restraint, and important conformational changes cannot be observed, so this refinement stage is essential. Comparing the results from 10a and 10b, demonstrates, that the MD cycles were necessary to find the correct position. Therefore, the $MD_B$ and $MD_{BSA}$, may be considered as "equilibration" stages, and were needed for the system to reach the energy minima, and properly hydrate both the ligand and the target. In some cases, a re-ordering of the initial ranks, based on the final $E_{LJ}$ promoted the correct binding sites (binding conformations) in higher ranks (1be9, 3g5d, 1qcf), in some cases, the ranking was not changed (3ptb, 2bal, 1hvy, 3cpa), but there were also a few cases, where the ranking worsened (3hvc, 3n3l, 1h61).

## Table S9. Detailed description of final Shaker protocol

**Methods**

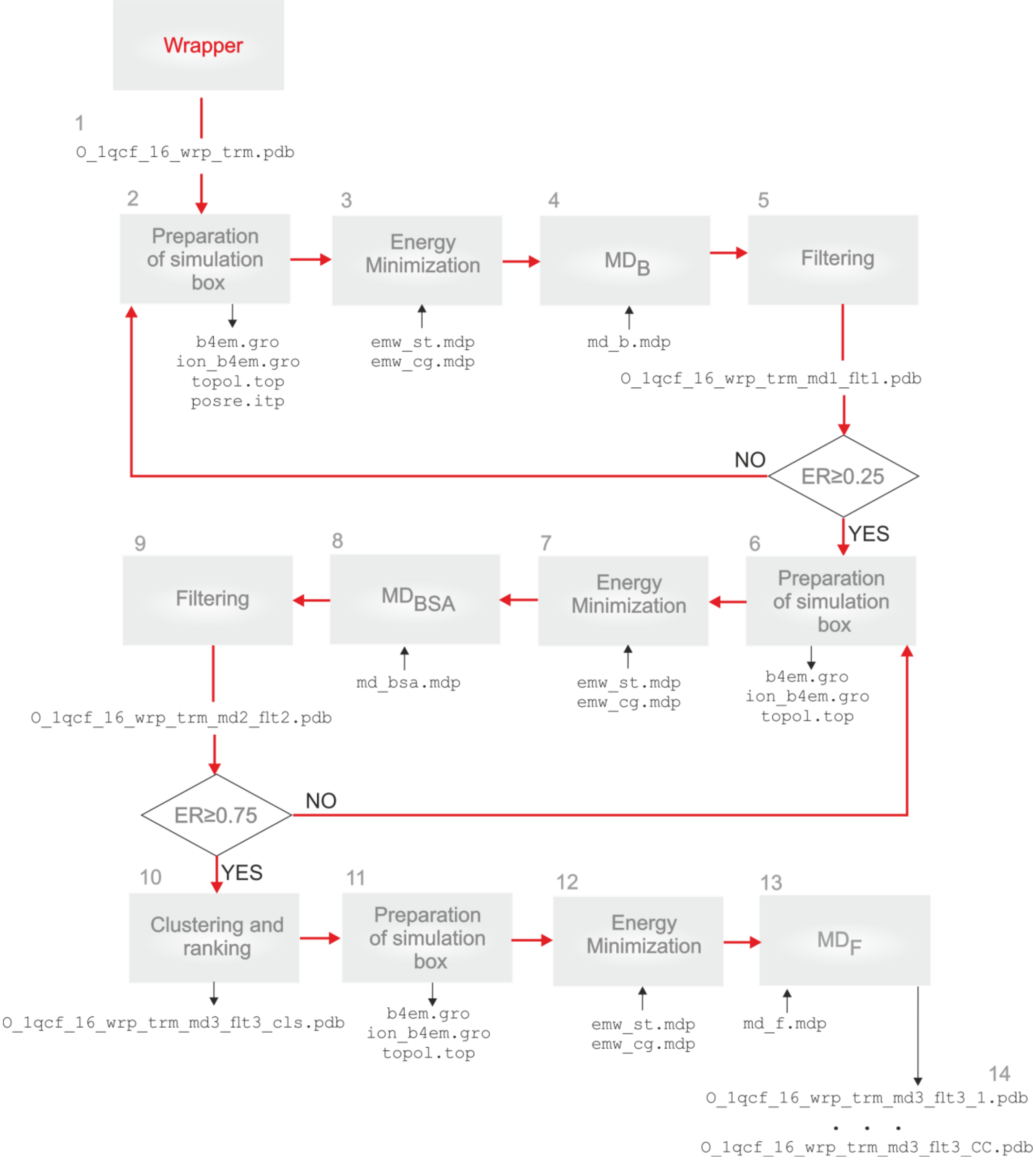


Input and output files names correspond to the example files, presented on web site associated to the WnS method (www.wnsdock.xyz). In the presented files 1qcf stands for the system name, and 16 for the total number of wrapper cycles performed.

**1 Inputs**

The target-ligand$_N$ pdb complex (O_1qcf_16_wrp_trm.pdb) resulted from the Wrapper step is subjected directly to the MD simulations.

**2 Preparation of the simulation box**

Conversion of the pdb files, into GROMACS input, and preparation of the simulation box were performed with pdb2gmx, editconf and solvate Gromacs commands. These steps were performed one time for the target-ligand$_N$ (N = the number of ligand copies resulted after Wrapper) complex. TIP3P water model, Amber99sb-ILDN force field and a cubic box with 10 Å (=1 nm) spacing from the solute were used (Table S1). For description of commands and input/output file types, please, refer to the GROMACS User's Manual.

```
gmx pdb2gmx -water tip3p -ff amber99sb-ildn -ignh –f O_1qcf_16_wrp_trm.pdb
gmx editconf -o -d 1 -bt cubic -f conf.gro
gmx solvate -cp out -cs -o b4em -p topol
gmx genrestr –f b4em –o posre.itp <<EOF
4
EOF
```

In cases where the target has non-zero net charge, neutrality of the system has to be ensured by adding the necessary number (X) of positive ($Na^+$) or negative ($Cl^-$) ions to the box, as Particle Mesh-Ewald (PME) summation was used for long range electrostatics. Gromacs command genion was used to add the necessary counter ions, by replacing the corresponding number of SOL molecules.

```
gmx grompp -v -f em_st -c b4em -o em_st -p topol
gmx genion -s em_st -o ion_b4em -p topol -pname NA -np X
gmx genion -s em_st -o ion_b4em -p topol -nname CL -nn X
```

**3 Energy minimization**

Before launching the production of the MD calculations, energy minimizations were performed on the box prepared at Step 2. A two-step minimization protocol was applied, including a steepest descent (st) and a conjugated gradient (cg) runs. Two scenarios were followed.

I) System with non-zero total charge (input: ion_b4em.gro file)

```
gmx grompp -v -f em_st -c ion_b4em -o st -p topol.top
gmx_d mdrun -v -s st -o st -c st -g st
gmx grompp -v -f em_cg -c st -o cg -p topol.top
gmx_d mdrun -v -s cg -o cg -c cg -g cg
```

II) System with zero total charge (input: b4em.gro file)

```
gmx grompp -v -f em_st -c b4em -o st -p topol.top
gmx_d mdrun -v -s st -o st -c st -g st
gmx grompp -v -f em_cg -c st -o cg -p topol.top
gmx_d mdrun -v -s cg -o cg -c cg -g cg
```

**4 $MD_B$**

MD simulations were launched using the energy minimized structure cg.gro as input file. $MD_B$ simulation parameters are specified in Table S1. Position restraints were defined in mdp file ("define = -DPOSRES"). The position restraints topology file (posre.itp) must be stored in the working directory, and used in the protein topology files, to ensure that backbone restraints are used instead of all heavy atoms restraint (eg.: in topol_Protein_chain_A.itp) Distance restraints were applied between the ion and the coordinating residues in order to maintain proper geometry, if the system contained coordinated structural ions (such as $Zn^{2+}$, Ni $^{2+}$, etc.).

```
gmx grompp -f md_b -o md_b -c cg -r cg -p topol.top -maxwarn 1
gmx mdrun -v -s O_1qcf_16_wrp_trm_md1.tpr -o md_b -c md_b -g md_b.log
```

The obtained trajectory file (md_b.trr), was converted to xtc file. The below succession of command lines performs this conversion of trr to xtc, handles the periodic boundary effects, centers the system in the box and fits the target molecules in subsequent frames on the top of the first frame.

```
gmx trjconv -f md_b.trr -s O_1qcf_16_wrp_trm_md1.tpr -o pbc_1.xtc -pbc whole << EOF
0
EOF
gmx trjconv -f pbc_1.xtc -s O_1qcf_16_wrp_trm_md1.tpr -o pbc_2.xtc -pbc cluster << EOF
1
0
EOF
gmx trjconv -f pbc_2.xtc -s O_1qcf_16_wrp_trm_md1.tpr -o pbc_3.xtc -center -pbc mol -ur compact
<< EOF
1
0
EOF
gmx  trjconv  -f  pbc_3.xtc  -s  O_1qcf_16_wrp_trm_md1.tpr  -o  O_1qcf_16_wrp_trm_md1.xtc  -fit
progressive << EOF
3
0
EOF
```

**5 Filtering**

The resulted xtc file was further processed by writing out the pdb files, for each simulation frame, using the command line below.

```
gmx trjconv –f O_1qcf_16_wrp_trm_1.xtc –s md_b.tpr –o O_1qcf_16_wrp_trm_md1_–sep << EOF
0
EOF
```

On the resulted 51 pdb structure (51 frames) of the trajectory, FS filtering method was applied (Table S4).

First two filters

The main purpose of these filters, is to calculate the smallest interatomic distance between the heavy atoms of the target and ligand molecules for each frame. This distance was calculated using an internal distance script, for all 51 frames. Using the distance values calculated with the distance script, two filtering steps were applied. Filtering was performed with an internal filtering script, as described in Table S4. Initially, ligands with large average distance from the target surface were filtered out. Finally, ligands not residing on the target surface at the final frame of the simulation ($51^{st}$ distance value) were removed. In both filtering steps, the maximal permitted heavy atoms distance between target-ligand was set to 3.5 Å.

Last two filters

The purpose of these filters was the elimination of weak binders, where the target-ligand binding energy increased more than 25% (from the initial frame) during the simulations. Such increase suggests an unstable binding pose and binding site. Using an energy script, LJ interaction was calculated between target and every ligand copy, during every frame of the simulation. LJ interaction calculation was performed using the equation described at Table S4. The energy filters were set up in two steps, using an energy filtering script, similarly to the distance filters. Initially we filter out ligands that have a weak binding interaction in average, during the MD simulations ($E(LJ)_{inter,\ AVG}$>25 % from first frame), and finally ligands that are binding weakly only at the final frame of the simulation ($E(LJ)_{inter,\ NF}$>25 % from first frame).
Elimination rate is checked after every FS. The $EC_S$ #1 (ER ≥ 0.25) is checked after $MD_B$. If the $EC_S$ #1 is met, the $MD_{BSA}$ simulation preparations are started. If the ER $EC_S$ #1 is not met, a second $MD_B$ cycle is performed.
**NOTE 1**: In all of our test systems, only one $MD_B$ was sufficient to reach the $EC_S$ #1, therefore in our test systems the above described steps (1-5), were always performed only once.
**NOTE 2:** In system 1 (3ptb), after the first $MD_B$ round, the $EC_S$ #2 was also met (ER for system 1: 0.91), and therefore in this case, no $MD_{BSA}$ cycle was performed. Thus, in case of System 1, after $MD_B$, Steps 5-8 were skipped, and after Step 4 (Filtering), step 9 (Clustering and ranking) was performed.

**6 Preparation of the simulation box**

O_1qcf_16_wrp_trm_md1_flt1.pdb file, resulted from Step 5 (filtering) is prepared for the next MD cycles, similarly to Step 1, except that in this Step, generation of target posre.itp is not necessary, because the one generated at Step 1, can be used here as well.

```
gmx pdb2gmx -water tip3p -ff amber99sb-ildn -ignh -f O_system_#cycle_wrp__md#_flt#.pdb
gmx editconf -o -d 1 -bt cubic -f conf.gro
gmx solvate -cp out -cs -o b4em -p topol
gmx grompp -v -f em_st -c b4em -o em_st -p topol
```

```
gmx genion -s em_st -o ion_b4em -p topol -pname NA -np X
gmx genion -s em_st -o ion_b4em -p topol -nname CL -nn X
```

**7 Energy minimization**

Before launching the production of the MD calculations, energy simulations were performed on the box prepared at Step 1. A two-step minimization protocol was applied, including a steepest descent (st) and a conjugated gradient (cg) runs. Two scenarios were followed.

I) System with non-zero total charge (input: ion_b4em.gro file)

```
gmx grompp -v -f em_st -c ion_b4em -o st -p topol.top
gmx_d mdrun -v -s st -o st -c st -g st
gmx grompp -v -f em_cg -c st -o cg -p topol.top
gmx_d mdrun -v -s cg -o cg -c cg -g cg
```

II) System with zero total charge (input: b4em.gro file)

```
gmx grompp -v -f em_st -c b4em -o st -p topol.top
gmx_d mdrun -v -s st -o st -c st -g st
gmx grompp -v -f em_cg -c st -o cg -p topol.top
gmx_d mdrun -v -s cg -o cg -c cg -g cg
```

**8 $MD_{BSA}$**

Using the energy minimized structure obtained from Step 7 (cg.gro) as input, MD calculation can be launched. $MD_{BSA}$ simulation parameters are specified in Table S1. Position restraints were defined in mdp file ("define = -DPOSRES"). The target position restraints topology file (posre.itp) must be stored in the working directory, and used in the protein topology files, to ensure that backbone restraints are used instead of all heavy atoms restraint (eg.: in topol_Protein_chain_A.itp) Distance restraints were applied between the ion and the coordinating residues in order to maintain proper geometry, if the System contains coordinated structural ions (such as $Zn^{2+}$, $Ni^{2+}$, etc.).

```
gmx grompp -f md_bsa -o O_1qcf_16_wrp_trm_md2_flt2 -c cg -r cg -p topol.top -maxwarn 1
gmx mdrun -v -s O_1qcf_16_wrp_trm_md2_flt2 -o md_bsa -c md_bsa -g md_bsa.log
```

The obtained trajectory file (md_bsa.trr), is converted to xtc file. The below succession of command lines performs this conversion of trr to xtc, handles the periodic boundary effects, centers the system in the box and fits the target molecules in subsequent frames on the top of the first frame.

```
gmx trjconv -f md_bsa.trr -s O_1qcf_16_wrp_trm_md2_flt2.tpr -o pbc_1.xtc -pbc whole << EOF
0
EOF
gmx trjconv -f pbc_1.xtc -s O_1qcf_16_wrp_trm_md2_flt2.tpr -o pbc_2.xtc -pbc cluster << EOF
1
0
EOF
gmx trjconv -f pbc_2.xtc -s O_1qcf_16_wrp_trm_md2_flt2.tpr -o pbc_3.xtc -center -pbc mol -ur
compact << EOF
1
```

```
0
EOF
gmx trjconv -f pbc_3.xtc -s O_1qcf_16_wrp_trm_md2_flt2.tpr -o O_1qcf_16_wrp_trm_md2_flt2.xtc -
fit progressive << EOF
3
0
EOF
```

**9 Filtering**

The filtering step, is applied, as described in Step 5, starting from the xtc as input file (O_1qcf_16_wrp_trm_md2_flt2.xtc). The only difference between this filtering and the one described at Step 5, is in the number of frames it is applied on. In Step 5, 51 frames were generated from the 5 ns $MD_B$ simulation, in this stage 201 frames were generated, as described in Table S1.

After the filtering step, ER check was performed, and if $EC_S$ #2 was met, the Step 10 was performed. If $EC_S$ #2 wasn't met, another $MD_{BSA}$ cycle starts, from Step 5. For the presented example (System 5 – 1qcf) two cycles of $MD_{BSA}$ were performed to reach the $EC_S$ #2.

**10 Clustering and ranking**

Clustering was performed on the pdb file resulted after the filtering step. The cluster representatives, are ranked based on the target-ligand LJ interaction calculated. The distance between the cluster representatives is set to a minimal 7 Å distance cut-off. RMSD calculations, were performed on the obtained conformations by comparing them to the reference X-ray structures (Table S10).

The resulted cluster representatives are split, and the target conformation is merged with each cluster representative, therefore resulting cluster count (CC) number of pdb structures. Each of these structures will be submitted to the following Steps (11-13), for a final structural refinement.

**11 Preparation of the simulation box**

The following steps are performed on each complex generated at Step 10. Preparation of the simulation box is similar to Step 2 and 6.

```
gmx pdb2gmx -water tip3p -ff amber99sb-ildn -ignh –f O_1qcf_16_wrp__md3_flt3_1.pdb
gmx editconf -o -d 1 -bt cubic -f conf.gro
gmx solvate -cp out -cs -o b4em -p topol
gmx grompp -v -f em_st -c b4em -o em_st -p topol
gmx genion -s em_st -o ion_b4em -p topol -pname NA -np X
gmx genion -s em_st -o ion_b4em -p topol -nname CL -nn X
```

**12 Energy minimization**

Energy minimization was performed similarly to Steps 3 and 7, for each complex obtained at Step 10.

**13 $MD_F$**

Using the energy minimized structure obtained at Step 12 (cg.gro), MD calculation can be launched. $MD_F$ simulation parameters are specified in Table S1. To ensure full flexibility on the system, ("define = -DFLEXIBLE") should be defined in the mdp file.

```
gmx grompp -f md_f -o O_1qcf_16_wrp_trm_md3_flt3_1.tpr -c cg -r cg -p topol.top -maxwarn 1
gmx mdrun -v -s O_1qcf_16_wrp_trm_md3_flt3_1.tpr -o md_f -c md_f -g md_f.log
```

The obtained trajectory file (md_f.trr), was converted to xtc file. The below succession of command lines performs this conversion of trr to xtc, handles the periodic boundary effects, centers the system in the box and fits the target molecules in subsequent frames on the top of the first frame.

```
gmx trjconv -f md_f.trr -s O_1qcf_16_wrp_trm_md3_flt3_1.tpr -o pbc_1.xtc -pbc whole << EOF
0
EOF
gmx trjconv -f pbc_1.xtc -s O_1qcf_16_wrp_trm_md3_flt3_1.tpr -o pbc_2.xtc -pbc cluster << EOF
1
0
EOF
gmx trjconv -f pbc_2.xtc -s O_1qcf_16_wrp_trm_md3_flt3_1.tpr -o pbc_3.xtc -center -pbc mol -ur
compact << EOF
1
0
EOF
gmx      trjconv      -f      pbc_3.xtc      -s      O_1qcf_16_wrp_trm_md3_flt3_1.tpr      -o
O_1qcf_16_wrp_trm_md3_flt3_1.xtc -fit progressive << EOF
3
0
EOF
```

The resulted xtc files were further processed for RMSD calculation, using the following Gromacs command. Following the instructions presented at Table S10 for each Test System, the alignment prior to RMSD calculation was done on the C$\alpha$ atoms, of the binding site residues (binding_site.ndx).

```
gmx rms –f O_1qcf_16_wrp_trm_1.xtc –s reference.pdb –o rmsd.xvg –what RMSD –n binding_site.ndx
–tu ns <<EOF
group number corresponding to the index group used (for alignment)
group number of the small ligand (for rmsd calculation)
EOF
```

**14 Outputs**

The pdb files, were extracted for each simulation frame (201 frames), for LJ interaction energy calculations using the command line below. The target-ligand LJ interaction energy

was calculated using an internal energy script, for each frame of the simulation. This calculated energy was used in the re-ranking results, presented at Table S8 and Appendix 3.

```
gmx trjconv -f O_1qcf_16_wrp_trm_md3_flt3_1.xtc -s O_1qcf_16_wrp_trm_md3_flt3_1.tpr -o O_1qcf_16_wrp_trm_md3_flt3_1_ -sep << EOF
0
EOF
```

## Table S10. Calculation of RMSD

| Methods |
|---|
| The root mean squared deviation (RMSD) of the calculated (C) ligand conformation from the experimental reference (R) conformation was calculated according to usual formula. $$RMSD_R = \sqrt{\frac{1}{NH_L}\sum_{i=0}^{NH_L}\left\|\vec{F}_{C,i} - \vec{R}_i\right\|^2}$$ where, $NH_L$ is the number of ligand heavy atoms, R is the space vector of the $i^{th}$ heavy atom of the reference ligand molecules, C is the space vector of the $i^{th}$ heavy atom of the calculated ligand conformation as resulted by the Shaker method. Two types of reference target−ligand complex structures (R= CR and MD), and the corresponding RMSDs are defined in the last paragraphs of text of this Table.<br><br>**Local alignment of target residues before RMSD calculation.** Similarly, to other studies (Dror, et al., 2011; Shan, et al., 2011) RMSD calculations were carried out using a local alignment on $C_\alpha$ atoms of the target residues forming the binding pocket, i.e. surrounding the reference ligand within a maximum 5 Å cut-off distance. The structural alignment was performed using GROMACS rms program (Abraham, et al., 2015). During flexible MD simulations, structural changes of the target can occur, rendering the comparison with the reference ligand structure difficult. With the above-mentioned local alignment of the holo and apo target structures using only the residues of the binding pocket, global structural changes of the target are disregarded and their influence on RMSD calculations is minimized. The list of the amino acids used for $C_\alpha$ alignment in RMSD calculation is presented below. |

| System Nr | PDB ID | Residues used for $C_\alpha$ alignment in RMSD calculation |
|---|---|---|
| 1a | 3PTB | D 172, S 173, C 174, Q 175, S 178, V 192, S 193, W 194, G 195, G 197, C 198, A 199, P 204, G 205, V 206, Y 207 |
| 2 | 3N3L | Y 10, K 57, N 59, R 60, T 63, S 205, F 206, P 209, F 239, L 344, K 347, I 348 |
| 3a | 3HVC | A 51, V 52, L 75, I 84, G 85, L 86, L 104, V 105, T 106, H 107, L 108, M 109, L 167, D 168 |
| 3b | 3HVC | M 194, L 195, W 197, H 228, I 229, L 232, S 252, S 254, A 255, R 256, Y 258 |
| 4 | 3CPA | H 69, E 72, R 127, D 142, N 144, R 145, H 196, S 197, Y 198, L 203, G 207, I 243, I 247, A 250, G 252, G 253, S 254, I 255, D 256, T 268, E 270, F 279 |
| 5 | 1QCF | L 273, V 281, A 293, K 295, E 310, M 314, T 338, E 339, Y 340, M 341, G 344, S 345, A 390, L 393, A 403, D 404 |
| 6 | 1H61 | T 26, Y 68, W 102, T 129, R 130, T 131, S 132, R 142, H 181, H 184, Y 186, Q 241, D 274, L 275, Y 351 |
| 7 | 2BAL | V 30, A 51, K 53, E 71, L 75, I 84, L 104, V 105, T 106, H 107, L 108, M 109, G 110, A 111, D 112, A 157, L 167 |

| | | |
|---|---|---|
| 8 | 1HVY | R 50, T 51, K 77, F 80, E 87, I 108, W 109, N 112, L 192, D 218, L 221, G 222, F 225, N 226, Y 258, I 307, M 309, M 311, A 312 |
| 9 | 3G5D | L 273, V 281, A 293, I 294, K 295, E 310, M 314, V 323, I 336, V 337, T 338, E 339, Y 340, M 341, S 342, K 343, G 344, L 393, A 403, D 404 |
| 10 | 1BE9 | R 14, G 18, L 19, G 20, F 21, N 22, I 23, I 24, G 25, S 35, H 68, A 72, L 75 |

**$RMSD_{CR}$.** Holo and apo target structures were structurally aligned on their backbone $\alpha$ carbon atoms ($C_\alpha$ alignment). Using the aligned structures, the crystalized ligand from the holo structure, was merged with the apo target structure, because the apo target was used in the wrapping process. The obtained ligand – apo target complex was used for $RMSD_{CR}$ calculations. In this reference structure the crystallographic ligand structure was used without any modifications, because this reference structure is most commonly used for RMSD calculations. RMSD values of this study are $RMSD_{CR}$ values except Tables S7 and S8, where $RMSD_{MD}$ was also used.

**$RMSD_{MD}$.** As it can be observed in Table S1, some of the investigated ligands have an increased B-factor (System 2), which suggests an increased mobility in the binding pocket. Additionally, in Table S8, we can observe serious RMSD variations which also suggest that ligands are not settled in a fix position in the binding site. Considering that the crystalized ligand can also move in the binding pocket, and is not fixed in the position that was crystallized we have performed short 10-ns MD on the ligand – apo target complex (see $RMSD_{CR}$), using the same MD parameters as in our $MD_B$ simulation (Table S1). The conformation of the ligand – apo target complex from the last frame of this MD, was used as reference structure, in our $RMSD_{MD}$ calculations.

**Table S11. Blind docking with association MD: A pilot study**

| **Goals** | | | |
|---|---|---|---|
| In a recent study (Shan, et al., 2011) MD simulations were used to follow the entire ligand binding process. In order to reproduce a similar binding pathway, we have started to work and analyze this new approach of blind docking. We studied if the ligand can find the binding site by association in reasonable time in case of β-trypsin – benzamidine complex. | | | |
| **Methods** | | | |
| Three pilot simulations were performed for the bovine β-trypsin – benzamidine complex (3ptb), in order to follow the entire binding process and produce correct binding poses of ligand molecules on their targets using explicit water models at atomic resolution. In the 1 μs–long simulations the ligand was set in three different starting positions, at various distances from the native, crystallographic binding site. | | | |
| **Results** | | | |
| Evaluating the results from the table below, we can observe that only the ligands that started the closest to the native pocket, were able to find the experimentally-observed conformation within 1 μs simulation. Additionally to the below table, Figure 3 (see main text) presents the followed binding pathways of the analyzed ligands. | | | |
| **Simulation nr** | **Starting dist. (Å)** | **Starting $RMSD_{CR}$ (Å)** | **Final $RMSD_{CR}$ (Å)** |
| 1 | 5.40 | 5.47 | 0.83 |
| 2 | 20.00 | 19.94 | 1.50 |
| 3 | 33.60 | 34.66 | 9.73 |
| | | | |
| **Conclusions** | | | |
| As it was explained in more details in the main text, one of the most important shortcoming of docking with MD simulations, is the lack of an exit condition. In other words, in cases where no information is known regarding the binding site of the ligand, it is hard to determine a priori the length of a simulation. | | | |

## Table S12. Computational times of Shaker

| System Nr. | PDB ID | Nr of Cycles until $EC_S$ #1[1] | Nr of Cycles until $EC_S$ #2[2] | Tot. Comp. time $MD_B$+$MD_{BSA}$(ns)[3] | Tot. Comp. time $MD_F$(ns)[4] |
|---|---|---|---|---|---|
| 1a | 3PTB | 1 | - | 5 | 120 |
| 1b | 3PTB | 1 | - | 5 | - |
| 1c | 3PTB | 1 | - | 5 | - |
| 2 | 3N3L | 1 | 1 | 25 | 260 |
| 3 | 3HVC | 1 | 1 | 25 | 420 |
| 4[6] | 3CPA | 1 | 2 | 45 | 240[6] |
| 5 | 1QCF | 1 | 2 | 45 | 240 |
| 6 | 1H61 | 1 | 2 | 45 | 240 |
| 7 | 2BAL | 1 | 2 | 45 | 240 |
| 8 | 1HVY | 1 | 2 | 45 | 200 |
| 9 | 3G5D | 1 | 6 | 125 | 200 |
| 10 | 1BE9 | 1 | 2 | 45 | 80 |

**Notes**

[1] Number of cycles necessary to achieve ER $EC_S$ # 1 after $MD_B$. Each simulation in a cycle was 5 ns long.

[2] Number of cycles necessary to achieve ER $EC_S$ # 2 after $MD_{BSA}$. Each simulation in a cycle was 20 ns long.

[3] Total computational time (ns) required to achieve $EC_S$ # 2.

**Tot. $MD_B$ + $MD_{BSA}$ time [3] = 5 Nr of cycles until** $EC_S$ **#1[1] + 20 Nr of cycles until $EC_S$ #2[2] (ns)**

[4] Total computational time (ns) required for system refinement.

**Tot. $MD_F$ time[4] = 20Total Clust Nr (ns)**

A 20-ns $MD_F$ simulation was performed for each cluster representative obtained. Please refer to Table 1 for the number of clusters obtained.

A 10-ns long simulation was performed for each ligand copy (N) obtained after wrapper step. Please refer to Table 2 in main text, and Appendix 3 for the number of N obtained.

[6] In this case, the refinement of the cluster representative with the lowest RMSD, was simulated for 100 ns instead of 20 ns. Please see Table S8: Results – RMSD

## Table S13. Effect of scoring on Wrapper results

| **Goals** | | | | | |
|---|---|---|---|---|---|
| In case of System 6, analyzing the ligand poses from the first cluster (AD4 scoring), we observed that a conformation that was closer to the reference structure was found within the cluster, but not as CR. Therefore, we performed a re-scoring experiment, with the purpose to check if the conformation closer to the reference structure could be the cluster representative or not, if different $E_{inter}$ is calculated. It was also a challenge to test if our Wrapper method (Table S3, new atom type) could be extended to other possible scoring functions or not. | | | | | |
| **Methods** | | | | | |
| We tested a re-scoring of the first wrapping cycle, for every analyzed system (see Appendix 4). Lennard-Jones potential was used instead of AutoDock 4.2 (AD4) scoring function(Morris, et al., 2009), and the hundreds of ligand conformations obtained with docking were clustered and ranked, based on the $E_{LJ}$ potential instead of the AD4 scoring. In case of System 6, not only the first wrapping cycle was rescored, but the whole wrapper step was performed using the calculated $E_{LJ}$ potential, instead of AD4 score. | | | | | |
| **Results** | | | | | |
| In the table below and in Appendix 4, the cluster numbers of the conformations with the lowest RMSD values can be observed. Only for System 6, this re-scoring induced an improvement in the RMSD, and this is the reason why only in this case, the wrapping was performed using the LJ potential instead of AD4 scoring function. For the rest of the systems, re-scoring was tested only for the first docking cycle, but since did not improved the RMSD values, the whole wrapper step was carried out using AD4 scoring function. | | | | | |
| **System Nr** | **PDB ID** | **Clusters with $E_{LJ}$ score** | **Clusters with AD4 score** | **RMSD with $E_{LJ}$ score (Å)** | **RMSD with AD4 score (Å)** |
| 1a | 3PTB | 1 | 1 | 4.3 | 0.3 |
| 2 | 3N3L | 2 | 1 | 3.8 | 3.9 |
| 3a | 3HVC | 2 | 4 | 8.1 | 8.9 |
| 3b | 3HVC | 5 | 8 | 20.1 | 23.8 |
| 4 | 3cpa | 1 | 1 | 3.4 | 3.4 |
| 5 | 1QCF | 7 | 4 | 1.6 | 1.6 |
| 6 | 1H61 | 1 | 1 | 3.5 | 6.8 |
| 7 | 2BAL | 3 | 7 | 10.0 | 3.9 |
| 8 | 1HVY | 1 | 1 | 3.5 | 3.5 |
| 9 | 3G5D | 3 | 2 | 2.8 | 1.3 |
| 10a | 1be9 | 1 | 1 | 7.3 | 6.6 |
| **Conclusion** | | | | | |
| Re-scoring with $E_{LJ}$ potential did not improve greatly the RMSD values for most of the analyzed systems, except in case of System 6. The improvement in case of System 6 was considerable (from 6.8 Å to 3.5 Å), and therefore the whole Wrapper step was performed using LJ potential | | | | | |

as the scoring function. Besides improving the RMSD in case of System 6, the fact that it was possible to carry out the whole wrapping process using LJ potential, instead of AD4 scoring, also demonstrates the robustness of the Wrapper method.

**Table S14. Shaker results after $MD_{BSA}$: 3ptb**

| **Goals** |
|---|
| Ligand conformations obtained after the clustering Step of the Shaker protocol. |
| **Results** |
| 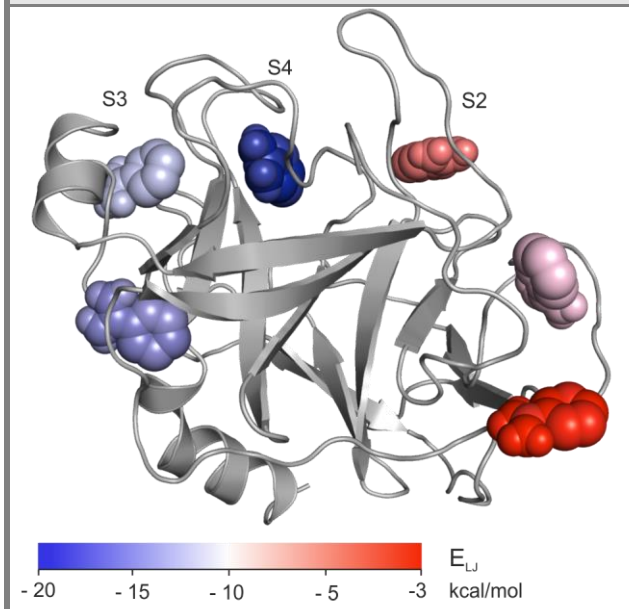  Color pallet (blue-white-red) of the ligands (spheres) indicates $E_{LJ}$ interaction (from strong to weak) between the ligands and protein (grey cartoon). The prerequisite binding sites, marked with S2 and S3, were reported in a previous study (Tiwary, et al., 2015), and we were able to find these additional binding sites, with the Shaker protocol. |

## Table S15. $MD_F$ results: 0 ns and 20 ns comparison (3ptb)

**Goals**

Analysis of binding pockets obtained from the Shaker protocol, before and after $MD_F$ refinement step.

**Results**

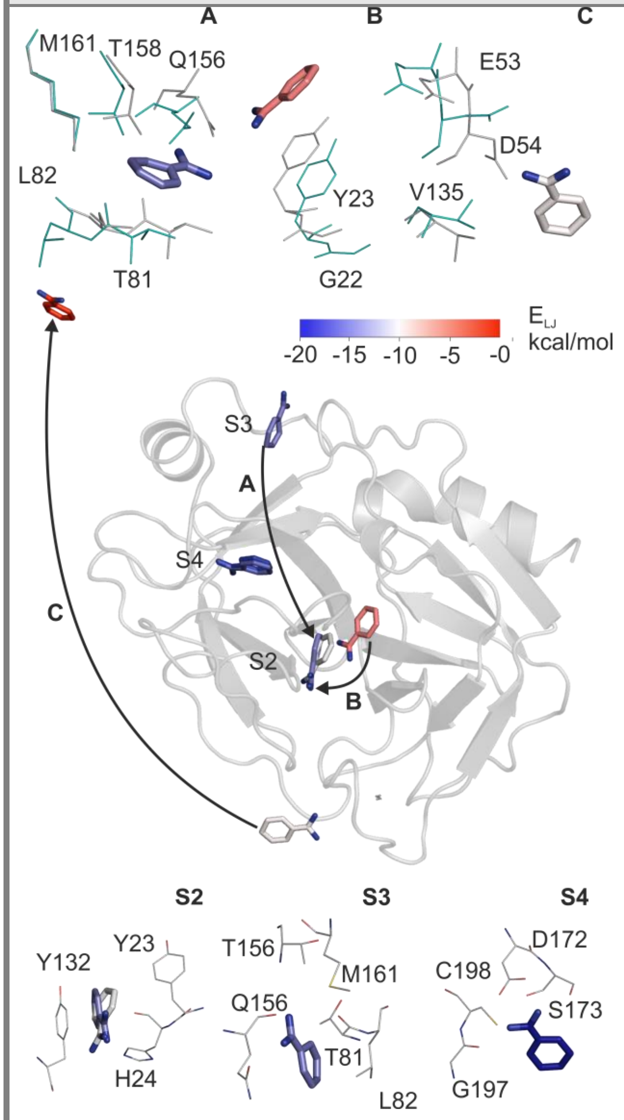


__*Top*__: benzamidine binding positions at 0 ns, before the $MD_F$ are presented with sticks, and color coded, based on the calculated $E_{LJ}$. Target residue positions at 0 ns (before the $MD_F$) are presented with grey lines, and residue positions at 20 ns (after $MD_F$), are colored with teal. This upper part of the figure captures the target residues that are responsible for ligand movements during the simulations.

__*Middle*__: the target (grey cartoon) is presented, with the main binding pocket (S4), and the previously reported sub pockets noted with S2, S3, of benzamidine. Ligands are colored, based on the calculated $E_{LJ}$ interaction energy with the target. Dark blue representing the strong

target-ligand interactions (-18 kcal/mol), while red color represents weak or no interaction (0 kcal/mol). Two binding (A and B) and one unbinding (C) pathways are presented.
*Bottom*: benzamidine binding conformations, in the three known binding pockets.

The impact of the target flexibility was analyzed after $MD_F$, on the resulted ligand conformations. During the flexible MD runs S4 binding pocket was the most stable. Benzamidine conformation bound initially to the S3 site, dissociated, and bound to S2 binding site during the $MD_F$ run (path A). In path A Ligand dissociation from S3, was possible, because of Q156, T81 and L82 movement in the binding pocket. S2 binding pocket was approached via path B as well, because π-π interaction between benzamidine and Y23 from the initial binding pocket was terminated during $MD_F$. In Path C, dissociations from the target were observed, due to target residue flexibility in the binding pocket (E53, D54, V135). These examples support the observations, that in ligand elimination, beside the water molecules target flexibility is also an important factor.

**Table S16. $MD_F$ results: 0 ns and 20 ns comparison (1qcf)**

| Goals |
| --- |
| Analysis of binding pockets, and ligand conformation changes before and after $MD_F$. |
| **Results** |
| 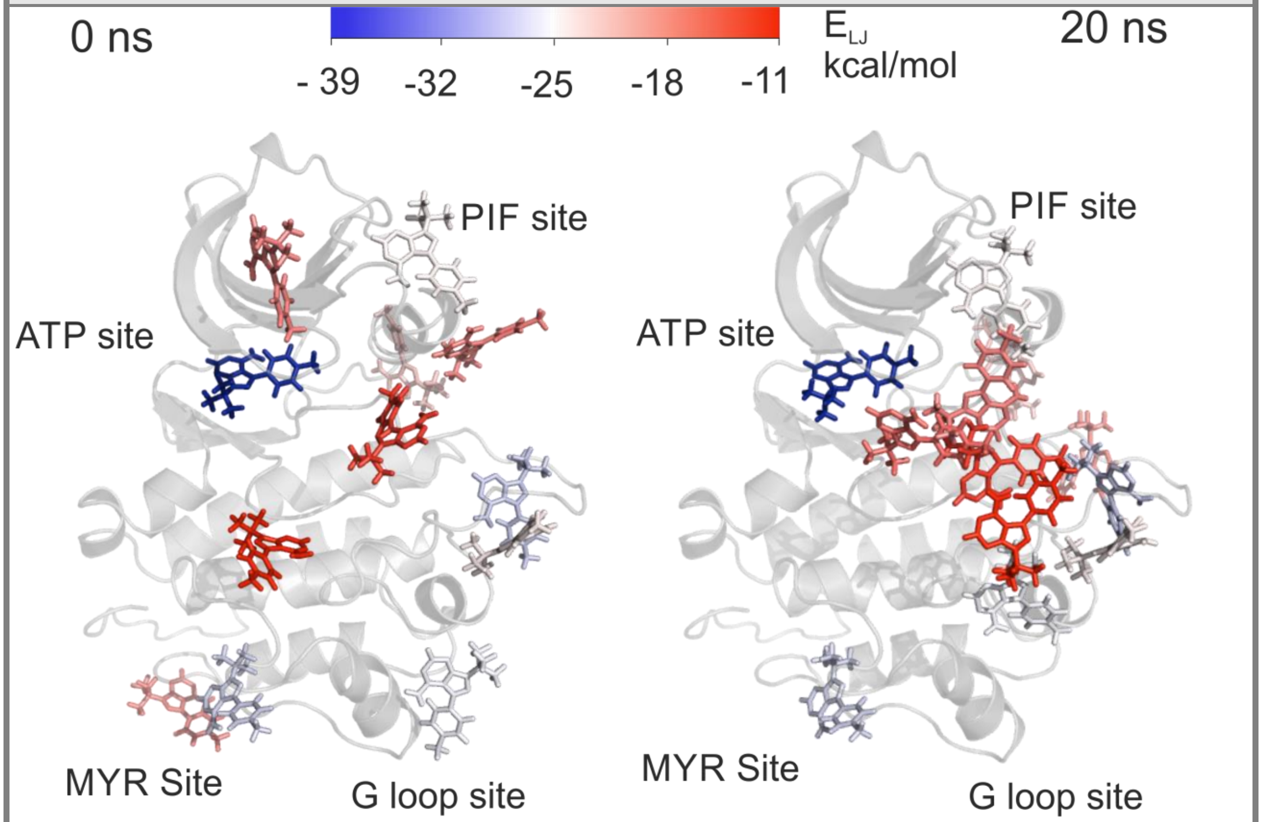  |
| Color pallet (blue-white-red) of the ligands (sticks) indicates calculated $E_{LJ}$ (from strong to weak) between the ligands and protein (grey cartoon). Ligand conformations at 0 ns (before $MD_F$) and 20 ns (after $MD_F$) are represented. For System 5, the previously reported sub pockets are noted with ATP, PIF, G-loop and MYR site.<br><br>Comparing the results at 0 and 20 ns of the $MD_F$ runs, it can be observed, that no ligand dissociated, in contrast to System 1, where ligand dissociation occurred (Table S15). In case of System 5, instead of dissociation, ligands migrated on the surface of the protein, from lower binding sites (MYR site, G-loop site), to stronger binding positions (ATP binding site and A loop). |

**Table S17. MD$_F$ results: Comparison of ligand conformations at 0 ns and 20 ns (3g5d)**

| Goals |
|---|
| Analysis of binding pockets from Shaker, and ligand movements obtained before and after MD$_F$ |
| **Results** |
| 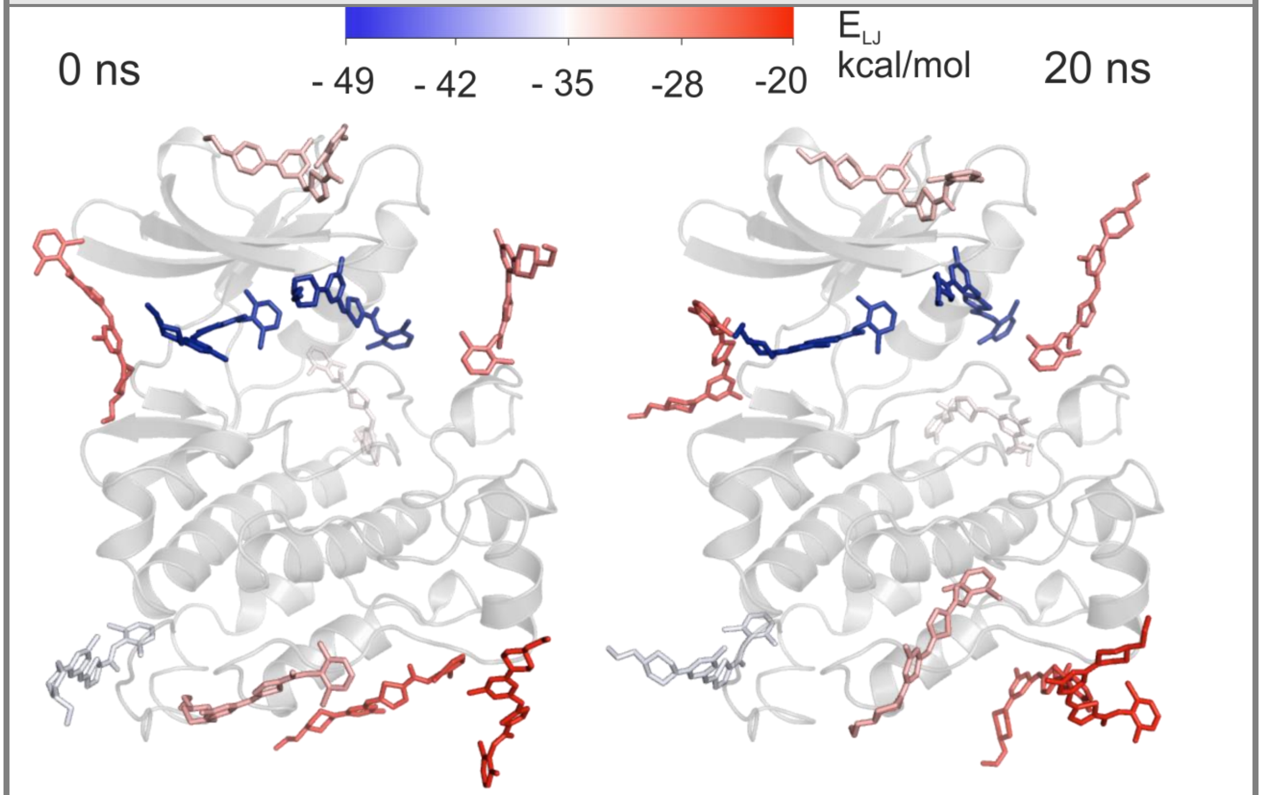  |
| Color pallet (blue-white-red) of the ligands (spheres) indicates $E_{LJ}$ interaction (from strong to weak) between the ligands and protein (grey cartoon). Ligand conformations before and after the refinement MD$_F$ runs are represented. |

## **Appendix** 1

Calculation of LJ potential, was performed using the General conditions and specific parameters listed in Table S3. Analyzing the obtained values for the three pairwise interactions selection of $R_X$ was performed.

**A Pairwise interaction: X – H.** Selection of $R_X$ when the reference atom is hydrogen. Considering the equilibrium distance between the X and H constant (2 Å), and $\varepsilon_X = 10^{-4}$ kcal/mol, the smallest $R_X$ value, for which a $V_{XY}$ was ≥ 1 kcal/mol, was 5 Å

| $R_X$ \ $\varepsilon_X$ | $10^{-5}$ | $2*10^{-5}$ | $3*10^{-5}$ | $4*10^{-5}$ | $5*10^{-5}$ | $6*10^{-5}$ | $7*10^{-5}$ | $8*10^{-5}$ | $9*10^{-5}$ | $10^{-4}$ | $10^{-3}$ |
|---|---|---|---|---|---|---|---|---|---|---|---|
| 2 | 0.00 | 0.00 | 0.00 | 0.00 | 0.00 | 0.00 | 0.00 | 0.00 | 0.00 | 0.00 | 0.00 |
| 2.1 | 0.00 | 0.00 | 0.00 | 0.00 | 0.00 | 0.00 | 0.00 | 0.00 | 0.00 | 0.00 | 0.00 |
| 2.2 | 0.00 | 0.00 | 0.00 | 0.00 | 0.00 | 0.00 | 0.00 | 0.00 | 0.00 | 0.00 | 0.00 |
| 2.3 | 0.00 | 0.00 | 0.00 | 0.00 | 0.00 | 0.00 | 0.00 | 0.00 | 0.00 | 0.00 | 0.00 |
| 2.4 | 0.00 | 0.00 | 0.00 | 0.00 | 0.00 | 0.00 | 0.00 | 0.00 | 0.00 | 0.00 | 0.00 |
| 2.5 | 0.00 | 0.00 | 0.00 | 0.00 | 0.00 | 0.00 | 0.00 | 0.00 | 0.00 | 0.00 | 0.00 |
| 2.6 | 0.00 | 0.00 | 0.00 | 0.00 | 0.00 | 0.00 | 0.00 | 0.00 | 0.00 | 0.00 | 0.00 |
| 2.7 | 0.00 | 0.00 | 0.00 | 0.00 | 0.00 | 0.00 | 0.00 | 0.00 | 0.00 | 0.00 | 0.01 |
| 2.8 | 0.00 | 0.00 | 0.00 | 0.00 | 0.00 | 0.00 | 0.00 | 0.00 | 0.00 | 0.00 | 0.01 |
| 2.9 | 0.00 | 0.00 | 0.00 | 0.00 | 0.00 | 0.01 | 0.01 | 0.01 | 0.01 | 0.01 | 0.02 |
| 3 | 0.00 | 0.00 | 0.01 | 0.01 | 0.01 | 0.01 | 0.01 | 0.01 | 0.01 | 0.01 | 0.03 |
| 3.1 | 0.00 | 0.01 | 0.01 | 0.01 | 0.01 | 0.01 | 0.01 | 0.01 | 0.01 | 0.01 | 0.04 |
| 3.2 | 0.01 | 0.01 | 0.01 | 0.01 | 0.01 | 0.01 | 0.02 | 0.02 | 0.02 | 0.02 | 0.06 |
| 3.3 | 0.01 | 0.01 | 0.01 | 0.02 | 0.02 | 0.02 | 0.02 | 0.02 | 0.02 | 0.03 | 0.08 |
| 3.4 | 0.01 | 0.02 | 0.02 | 0.02 | 0.02 | 0.03 | 0.03 | 0.03 | 0.03 | 0.03 | 0.11 |
| 3.5 | 0.01 | 0.02 | 0.02 | 0.03 | 0.03 | 0.04 | 0.04 | 0.04 | 0.04 | 0.05 | 0.14 |
| 3.6 | 0.02 | 0.03 | 0.03 | 0.04 | 0.04 | 0.05 | 0.05 | 0.05 | 0.06 | 0.06 | 0.19 |
| 3.7 | 0.02 | 0.03 | 0.04 | 0.05 | 0.05 | 0.06 | 0.06 | 0.07 | 0.07 | 0.08 | 0.24 |
| 3.8 | 0.03 | 0.04 | 0.05 | 0.06 | 0.07 | 0.07 | 0.08 | 0.09 | 0.09 | 0.10 | 0.30 |
| 3.9 | 0.04 | 0.05 | 0.07 | 0.08 | 0.09 | 0.09 | 0.10 | 0.11 | 0.11 | 0.12 | 0.38 |
| 4 | 0.05 | 0.07 | 0.08 | 0.10 | 0.11 | 0.12 | 0.13 | 0.14 | 0.14 | 0.15 | 0.48 |
| 4.1 | 0.06 | 0.08 | 0.10 | 0.12 | 0.13 | 0.15 | 0.16 | 0.17 | 0.18 | 0.19 | 0.60 |
| 4.2 | 0.07 | 0.10 | 0.13 | 0.15 | 0.16 | 0.18 | 0.19 | 0.21 | 0.22 | 0.23 | 0.74 |
| 4.3 | 0.09 | 0.13 | 0.16 | 0.18 | 0.20 | 0.22 | 0.24 | 0.26 | 0.27 | 0.29 | 0.91 |
| 4.4 | 0.11 | 0.16 | 0.19 | 0.22 | 0.25 | 0.27 | 0.29 | 0.31 | 0.33 | 0.35 | 1.11 |
| 4.5 | 0.14 | 0.19 | 0.23 | 0.27 | 0.30 | 0.33 | 0.36 | 0.38 | 0.41 | 0.43 | 1.35 |
| 4.6 | 0.16 | 0.23 | 0.28 | 0.33 | 0.37 | 0.40 | 0.43 | 0.46 | 0.49 | 0.52 | 1.64 |
| 4.7 | 0.20 | 0.28 | 0.34 | 0.40 | 0.44 | 0.49 | 0.52 | 0.56 | 0.60 | 0.63 | 1.98 |
| 4.8 | 0.24 | 0.34 | 0.41 | 0.48 | 0.53 | 0.59 | 0.63 | 0.68 | 0.72 | 0.76 | 2.39 |
| 4.9 | 0.29 | 0.41 | 0.50 | 0.57 | 0.64 | 0.70 | 0.76 | 0.81 | 0.86 | 0.91 | 2.87 |
| 5 | 0.34 | 0.49 | 0.59 | 0.69 | 0.77 | 0.84 | 0.91 | 0.97 | 1.03 | 1.09 | 3.43 |

**B Pairwise interaction: X – C.** Selection of Rx when the reference atom is carbon. Considering the equilibrium distance between the X and C constant (2 Å), and epsilon = $10^{-4}$ kcal/mol, the smallest Rx value, for which a $V_{XY} \geq 1$ kcal/mol, was 2.5 Å

| $\varepsilon_X$ / $R_X$ | $10^{-5}$ | $2*10^{-5}$ | $3*10^{-5}$ | $4*10^{-5}$ | $5*10^{-5}$ | $6*10^{-5}$ | $7*10^{-5}$ | $8*10^{-5}$ | $9*10^{-5}$ | $10^{-4}$ | $10^{-3}$ |
|---|---|---|---|---|---|---|---|---|---|---|---|
| 2 | 0.13 | 0.19 | 0.23 | 0.26 | 0.29 | 0.32 | 0.35 | 0.37 | 0.39 | 0.41 | 1.31 |
| 2.1 | 0.16 | 0.23 | 0.28 | 0.33 | 0.36 | 0.40 | 0.43 | 0.46 | 0.49 | 0.52 | 1.63 |
| 2.2 | 0.20 | 0.29 | 0.35 | 0.40 | 0.45 | 0.49 | 0.53 | 0.57 | 0.60 | 0.64 | 2.02 |
| 2.3 | 0.25 | 0.35 | 0.43 | 0.50 | 0.55 | 0.61 | 0.66 | 0.70 | 0.74 | 0.78 | 2.48 |
| 2.4 | 0.30 | 0.43 | 0.53 | 0.61 | 0.68 | 0.74 | 0.80 | 0.86 | 0.91 | 0.96 | 3.04 |
| 2.5 | 0.37 | 0.52 | 0.64 | 0.74 | 0.83 | 0.91 | 0.98 | 1.05 | 1.11 | 1.17 | 3.70 |
| 2.6 | 0.45 | 0.64 | 0.78 | 0.90 | 1.00 | 1.10 | 1.19 | 1.27 | 1.35 | 1.42 | 4.49 |
| 2.7 | 0.54 | 0.77 | 0.94 | 1.09 | 1.21 | 1.33 | 1.44 | 1.54 | 1.63 | 1.72 | 5.43 |
| 2.8 | 0.65 | 0.93 | 1.13 | 1.31 | 1.46 | 1.60 | 1.73 | 1.85 | 1.96 | 2.07 | 6.54 |
| 2.9 | 0.79 | 1.11 | 1.36 | 1.57 | 1.76 | 1.92 | 2.08 | 2.22 | 2.36 | 2.48 | 7.86 |
| 3 | 0.94 | 1.33 | 1.63 | 1.88 | 2.10 | 2.30 | 2.49 | 2.66 | 2.82 | 2.97 | 9.40 |
| 3.1 | 1.12 | 1.59 | 1.94 | 2.24 | 2.51 | 2.75 | 2.97 | 3.17 | 3.36 | 3.55 | 11.21 |
| 3.2 | 1.33 | 1.89 | 2.31 | 2.67 | 2.98 | 3.27 | 3.53 | 3.77 | 4.00 | 4.22 | 13.34 |
| 3.3 | 1.58 | 2.24 | 2.74 | 3.16 | 3.54 | 3.87 | 4.18 | 4.47 | 4.74 | 5.00 | 15.81 |
| 3.4 | 1.87 | 2.64 | 3.24 | 3.74 | 4.18 | 4.58 | 4.95 | 5.29 | 5.61 | 5.91 | 18.70 |
| 3.5 | 2.21 | 3.12 | 3.82 | 4.41 | 4.93 | 5.40 | 5.84 | 6.24 | 6.62 | 6.98 | 22.06 |
| 3.6 | 2.60 | 3.67 | 4.50 | 5.19 | 5.80 | 6.36 | 6.87 | 7.34 | 7.79 | 8.21 | 25.96 |
| 3.7 | 3.05 | 4.31 | 5.28 | 6.09 | 6.81 | 7.46 | 8.06 | 8.62 | 9.14 | 9.63 | 30.46 |
| 3.8 | 3.57 | 5.05 | 6.18 | 7.14 | 7.98 | 8.74 | 9.44 | 10.09 | 10.70 | 11.28 | 35.68 |
| 3.9 | 4.17 | 5.89 | 7.22 | 8.34 | 9.32 | 10.21 | 11.03 | 11.79 | 12.51 | 13.18 | 41.68 |
| 4 | 4.86 | 6.87 | 8.42 | 9.72 | 10.87 | 11.90 | 12.86 | 13.75 | 14.58 | 15.37 | 48.60 |
| 4.1 | 5.65 | 8.00 | 9.79 | 11.31 | 12.64 | 13.85 | 14.96 | 15.99 | 16.96 | 17.88 | 56.54 |
| 4.2 | 6.56 | 9.28 | 11.37 | 13.13 | 14.68 | 16.08 | 17.37 | 18.57 | 19.69 | 20.76 | 65.65 |
| 4.3 | 7.61 | 10.76 | 13.18 | 15.22 | 17.01 | 18.63 | 20.13 | 21.52 | 22.82 | 24.06 | 76.08 |
| 4.4 | 8.80 | 12.44 | 15.24 | 17.60 | 19.68 | 21.55 | 23.28 | 24.89 | 26.40 | 27.82 | 87.99 |
| 4.5 | 10.16 | 14.37 | 17.59 | 20.32 | 22.71 | 24.88 | 26.88 | 28.73 | 30.47 | 32.12 | 101.58 |
| 4.6 | 11.71 | 16.56 | 20.28 | 23.41 | 26.18 | 28.67 | 30.97 | 33.11 | 35.12 | 37.02 | 117.06 |
| 4.7 | 13.47 | 19.05 | 23.33 | 26.93 | 30.11 | 32.99 | 35.63 | 38.09 | 40.40 | 42.59 | 134.67 |
| 4.8 | 15.47 | 21.87 | 26.79 | 30.93 | 34.58 | 37.88 | 40.92 | 43.75 | 46.40 | 48.91 | 154.66 |
| 4.9 | 17.73 | 25.08 | 30.71 | 35.47 | 39.65 | 43.44 | 46.92 | 50.16 | 53.20 | 56.08 | 177.33 |
| 5 | 20.30 | 28.71 | 35.16 | 40.60 | 45.39 | 49.72 | 53.71 | 57.42 | 60.90 | 64.19 | 203.00 |

**C Pairwise interaction: X – O.** Selection of Rx when the reference atom is oxygen. Considering the equilibrium distance between the X and O constant (2 Å), and epsilon = $10^{-4}$ kcal/mol, the smallest Rx value, for which a $V_{Xy} \geq 1$ kcal/mol, was 3.2 Å

| $\varepsilon_X$ / $R_X$ | $10^{-5}$ | $2*10^{-5}$ | $3*10^{-5}$ | $4*10^{-5}$ | $5*10^{-5}$ | $6*10^{-5}$ | $7*10^{-5}$ | $8*10^{-5}$ | $9*10^{-5}$ | $10^{-4}$ | $10^{-3}$ |
|---|---|---|---|---|---|---|---|---|---|---|---|
| 2 | 0.02 | 0.03 | 0.03 | 0.04 | 0.04 | 0.05 | 0.05 | 0.05 | 0.06 | 0.06 | 0.19 |
| 2.1 | 0.03 | 0.04 | 0.05 | 0.05 | 0.06 | 0.06 | 0.07 | 0.07 | 0.08 | 0.08 | 0.26 |
| 2.2 | 0.03 | 0.05 | 0.06 | 0.07 | 0.08 | 0.08 | 0.09 | 0.10 | 0.10 | 0.11 | 0.35 |
| 2.3 | 0.05 | 0.06 | 0.08 | 0.09 | 0.10 | 0.11 | 0.12 | 0.13 | 0.14 | 0.14 | 0.45 |
| 2.4 | 0.06 | 0.08 | 0.10 | 0.12 | 0.13 | 0.14 | 0.16 | 0.17 | 0.18 | 0.19 | 0.59 |
| 2.5 | 0.08 | 0.11 | 0.13 | 0.15 | 0.17 | 0.18 | 0.20 | 0.21 | 0.23 | 0.24 | 0.75 |
| 2.6 | 0.10 | 0.14 | 0.17 | 0.19 | 0.21 | 0.23 | 0.25 | 0.27 | 0.29 | 0.30 | 0.96 |
| 2.7 | 0.12 | 0.17 | 0.21 | 0.24 | 0.27 | 0.30 | 0.32 | 0.34 | 0.36 | 0.38 | 1.21 |
| 2.8 | 0.15 | 0.21 | 0.26 | 0.30 | 0.34 | 0.37 | 0.40 | 0.43 | 0.45 | 0.48 | 1.51 |
| 2.9 | 0.19 | 0.27 | 0.33 | 0.38 | 0.42 | 0.46 | 0.50 | 0.53 | 0.56 | 0.60 | 1.88 |
| 3 | 0.23 | 0.33 | 0.40 | 0.47 | 0.52 | 0.57 | 0.62 | 0.66 | 0.70 | 0.74 | 2.33 |
| 3.1 | 0.29 | 0.40 | 0.50 | 0.57 | 0.64 | 0.70 | 0.76 | 0.81 | 0.86 | 0.91 | 2.86 |
| 3.2 | 0.35 | 0.50 | 0.61 | 0.70 | 0.78 | 0.86 | 0.93 | 0.99 | 1.05 | 1.11 | 3.51 |
| 3.3 | 0.43 | 0.60 | 0.74 | 0.85 | 0.96 | 1.05 | 1.13 | 1.21 | 1.28 | 1.35 | 4.27 |
| 3.4 | 0.52 | 0.73 | 0.90 | 1.04 | 1.16 | 1.27 | 1.37 | 1.47 | 1.56 | 1.64 | 5.19 |
| 3.5 | 0.63 | 0.89 | 1.09 | 1.25 | 1.40 | 1.54 | 1.66 | 1.77 | 1.88 | 1.98 | 6.27 |
| 3.6 | 0.76 | 1.07 | 1.31 | 1.51 | 1.69 | 1.85 | 2.00 | 2.14 | 2.27 | 2.39 | 7.56 |
| 3.7 | 0.91 | 1.28 | 1.57 | 1.81 | 2.03 | 2.22 | 2.40 | 2.57 | 2.72 | 2.87 | 9.07 |
| 3.8 | 1.09 | 1.54 | 1.88 | 2.17 | 2.43 | 2.66 | 2.87 | 3.07 | 3.26 | 3.43 | 10.85 |
| 3.9 | 1.29 | 1.83 | 2.24 | 2.59 | 2.90 | 3.17 | 3.43 | 3.66 | 3.88 | 4.09 | 12.95 |
| 4 | 1.54 | 2.18 | 2.67 | 3.08 | 3.44 | 3.77 | 4.07 | 4.36 | 4.62 | 4.87 | 15.40 |
| 4.1 | 1.83 | 2.58 | 3.16 | 3.65 | 4.08 | 4.47 | 4.83 | 5.16 | 5.48 | 5.77 | 18.26 |
| 4.2 | 2.16 | 3.05 | 3.74 | 4.32 | 4.83 | 5.29 | 5.71 | 6.11 | 6.48 | 6.83 | 21.59 |
| 4.3 | 2.55 | 3.60 | 4.41 | 5.09 | 5.70 | 6.24 | 6.74 | 7.20 | 7.64 | 8.06 | 25.47 |
| 4.4 | 3.00 | 4.24 | 5.19 | 5.99 | 6.70 | 7.34 | 7.93 | 8.48 | 8.99 | 9.48 | 29.97 |
| 4.5 | 3.52 | 4.97 | 6.09 | 7.04 | 7.87 | 8.62 | 9.31 | 9.95 | 10.55 | 11.12 | 35.18 |
| 4.6 | 4.12 | 5.83 | 7.14 | 8.24 | 9.21 | 10.09 | 10.90 | 11.65 | 12.36 | 13.03 | 41.19 |
| 4.7 | 4.81 | 6.81 | 8.34 | 9.63 | 10.76 | 11.79 | 12.73 | 13.61 | 14.44 | 15.22 | 48.13 |
| 4.8 | 5.61 | 7.94 | 9.72 | 11.22 | 12.55 | 13.75 | 14.85 | 15.87 | 16.83 | 17.75 | 56.12 |
| 4.9 | 6.53 | 9.23 | 11.31 | 13.06 | 14.60 | 15.99 | 17.27 | 18.47 | 19.59 | 20.65 | 65.29 |
| 5 | 7.58 | 10.72 | 13.13 | 15.16 | 16.95 | 18.57 | 20.06 | 21.44 | 22.74 | 23.97 | 75.81 |

## Appendix 2

Systems used in Wrapper step are represented, with detailed Wrapper results.

| #System | PDB ID | Wrapper Cycles | $E_{inter}$[1] | ASA[2] | Total Nr of ligands[3] | N[4] |
|---|---|---|---|---|---|---|
| 1a | 3PTB | 1 | -6.2 | 99.90 | 1 | 68 |
| | | 2 | -4.1 | 96.73 | 4 | |
| | | 3 | -3.4 | 88.70 | 12 | |
| | | 4 | -2.6 | 79.78 | 21 | |
| | | 5 | -2.28 | 68.26 | 31 | |
| | | 6 | -1.67 | 65.06 | 35 | |
| | | 7 | -1.33 | 61.16 | 39 | |
| | | 8 | -0.96 | 51.18 | 48 | |
| | | 9 | -0.76 | 43.77 | 56 | |
| | | 10 | -0.44 | 37.46 | 63 | |
| | | 11 | 0.09 | 32.43 | 68 | |
| 1b | 3PTB | 1 | -6.2 | 99.88 | 1 | 74 |
| | | 2 | -4.11 | 97.88 | 3 | |
| | | 3 | -3.45 | 90.86 | 9 | |
| | | 4 | -2.93 | 80.47 | 19 | |
| | | 5 | -2.29 | 68.18 | 31 | |
| | | 6 | -1.59 | 63.79 | 36 | |
| | | 7 | -1.19 | 54.56 | 43 | |
| | | 8 | -0.76 | 42.64 | 55 | |
| | | 9 | -0.41 | 37.15 | 62 | |
| | | 10 | -0.1 | 32.44 | 68 | |
| | | 11 | **0.29** | 26.72 | 74 | |
| 1c | 3PTB | 1 | -6.2 | 99.89 | 1 | 71 |
| | | 2 | -4.1 | 96.44 | 4 | |
| | | 3 | -3.4 | 90.58 | 10 | |
| | | 4 | -2.9 | 78.95 | 21 | |
| | | 5 | -2.22 | 69.91 | 29 | |
| | | 6 | -1.79 | 64.10 | 35 | |
| | | 7 | -1.21 | 55.21 | 42 | |
| | | 8 | -0.9 | 43.72 | 52 | |
| | | 9 | -0.53 | 37.84 | 59 | |
| | | 10 | -0.24 | 28.75 | 68 | |
| | | 11 | **0.34** | 25.90 | 73 | |
| 2 | 3N3L | 1 | -7.24 | 96.97 | 6 | 300 |
| | | 2 | -5.96 | 92.96 | 12 | |
| | | 3 | -6.53 | 82.65 | 27 | |
| | | 4 | -5.65 | 77.47 | 34 | |
| | | 5 | -5.67 | 66.81 | 46 | |
| | | 6 | -5.48 | 57.24 | 62 | |
| | | 7 | -5.57 | 51.81 | 69 | |

| | | | | | | |
|---|---|---|---|---|---|---|
| | | 8 | -5.68 | 45.69 | 79 | |
| | | 9 | -5.14 | 35.36 | 96 | |
| | | 10 | -5.2 | 32.19 | 104 | |
| | | 11 | -5.53 | 27.45 | 115 | |
| | | 12 | -4.63 | 21.84 | 130 | |
| | | 13 | -4.47 | 16.85 | 142 | |
| | | 14 | -4.44 | 13.71 | 154 | |
| | | 15 | -4.61 | 10.25 | 162 | |
| | | 16 | -4.08 | 8.55 | 169 | |
| | | 17 | -3.77 | 8.12 | 175 | |
| | | 18 | -3.88 | 7.05 | 186 | |
| | | 19 | -3.47 | 5.74 | 198 | |
| | | 20 | -4.1 | 4.79 | 210 | |
| | | 21 | -3.39 | 3.63 | 217 | |
| | | 22 | -3.33 | 2.90 | 225 | |
| | | 23 | -3 | 2.54 | 234 | |
| | | 24 | -2.94 | 2.41 | 241 | |
| | | 25 | -7.24 | 2.33 | 250 | |
| | | 26 | -5.96 | 1.86 | 261 | |
| | | 27 | -6.53 | 1.46 | 271 | |
| | | 28 | -5.65 | 1.40 | 281 | |
| | | 29 | -5.67 | 1.03 | 292 | |
| | | 30 | -5.48 | 1.03 | 298 | |
| | | 31 | -5.57 | 1.03 | 307 | |
| | | 32 | -5.68 | ***1.00*** | 317 | |
| | | 1 | -6.83 | 96.43 | 8 | |
| | | 2 | -5.67 | 88.85 | 17 | |
| | | 3 | -5.09 | 76.80 | 30 | |
| | | 4 | -4.86 | 67.53 | 39 | |
| | | 5 | -4.37 | 56.39 | 51 | |
| | | 6 | -3.91 | 48.80 | 61 | |
| | | 7 | -3.49 | 43.57 | 68 | |
| | | 8 | -3.25 | 35.30 | 76 | |
| | | 9 | -3.03 | 30.68 | 86 | |
| 3 | 3HVC | 10 | -3.13 | 22.71 | 95 | 222 |
| | | 11 | -2.26 | 19.16 | 102 | |
| | | 12 | -2.17 | 14.57 | 115 | |
| | | 13 | -1.97 | 12.32 | 123 | |
| | | 14 | -1.69 | 8.33 | 136 | |
| | | 15 | -1.46 | 6.53 | 144 | |
| | | 16 | -1.34 | 5.87 | 148 | |
| | | 17 | -1.13 | 4.37 | 157 | |
| | | 18 | -1.1 | 2.90 | 172 | |
| | | 19 | -1.05 | 2.50 | 181 | |

| | | | | | | |
|---|---|---|---|---|---|---|
| | | 20 | -0.84 | 1.82 | 188 | |
| | | 21 | -0.69 | 1.57 | 200 | |
| | | 22 | -0.64 | 1.34 | 208 | |
| | | 23 | -0.6 | 1.17 | 215 | |
| | | 24 | -0.56 | **0.83** | 222 | |
| 4 | 3CPA | 1 | -8.83 | 77.03 | 17 | 155 |
| | | 2 | -6.59 | 54.18 | 34 | |
| | | 3 | -5.93 | 29.66 | 54 | |
| | | 4 | -5.08 | 22.80 | 66 | |
| | | 5 | -4.45 | 13.77 | 85 | |
| | | 6 | -3.77 | 8.77 | 98 | |
| | | 7 | -3.06 | 6.69 | 107 | |
| | | 8 | -3.01 | 2.89 | 124 | |
| | | 9 | -2.18 | 1.53 | 140 | |
| | | 10 | -2.22 | ***0.94*** | 155 | |
| 5 | 1QCF | 1 | -7.17 | 93.87 | 7 | 143 |
| | | 2 | -6.62 | 76.53 | 19 | |
| | | 3 | -5.71 | 65.31 | 27 | |
| | | 4 | -4.91 | 49.70 | 39 | |
| | | 5 | -4.25 | 38.97 | 48 | |
| | | 6 | -3.97 | 30.56 | 55 | |
| | | 7 | -3.77 | 21.02 | 65 | |
| | | 8 | -3.11 | 16.09 | 74 | |
| | | 9 | -2.85 | 12.88 | 82 | |
| | | 10 | -2.46 | 8.58 | 94 | |
| | | 11 | -1.86 | 6.04 | 101 | |
| | | 12 | -1.64 | 3.25 | 114 | |
| | | 13 | -1.44 | 2.34 | 125 | |
| | | 14 | -1.14 | 1.41 | 135 | |
| | | 15 | -0.81 | **0.82** | 143 | |
| 6 | 1H61 | 1 | -30.6 | 87.26 | 8 | 116 |
| | | 2 | -22.03 | 82.96 | 17 | |
| | | 3 | -20.02 | 49.41 | 31 | |
| | | 4 | -21.18 | 36.10 | 40 | |
| | | 5 | -13.41 | 26.60 | 48 | |
| | | 6 | -15.07 | 20.15 | 55 | |
| | | 7 | -15.73 | 13.60 | 63 | |
| | | 8 | -12.99 | 9.37 | 70 | |
| | | 9 | -8.88 | 5.87 | 79 | |
| | | 10 | -8.27 | 2.91 | 88 | |
| | | 11 | -1.96 | 1.60 | 100 | |
| | | 12 | -6.37 | 1.24 | 105 | |
| | | 13 | -9.31 | ***0.33*** | 116 | |
| 7 | 2BAL | 2 | -10.18 | 52.38 | 32 | 122 |

| | | | | | | |
|---|---|---|---|---|---|---|
| | | 3 | -7.95 | 32.88 | 45 | |
| | | 4 | -6.64 | 21.54 | 56 | |
| | | 5 | -4.91 | 14.70 | 66 | |
| | | 6 | -3.88 | 10.30 | 75 | |
| | | 7 | -2.41 | 5.86 | 84 | |
| | | 8 | -1.19 | 3.89 | 91 | |
| | | 9 | -0.64 | 2.61 | 109 | |
| | | 10 | ***0.2*** | 1.92 | 125 | |
| 8 | 1HVY | 1 | -14.6 | 78.47 | 10 | 106 |
| | | 2 | -10.89 | 58.84 | 20 | |
| | | 3 | -10.67 | 35.12 | 33 | |
| | | 4 | -11.01 | 16.93 | 47 | |
| | | 5 | -9.84 | 10.52 | 55 | |
| | | 6 | -7.99 | 7.10 | 62 | |
| | | 7 | -7.49 | 3.91 | 75 | |
| | | 8 | -6.95 | 2.39 | 83 | |
| | | 9 | -5.9 | 2.14 | 93 | |
| | | 10 | -5.75 | ***0.23*** | 106 | |
| 9 | 3G5D | 1 | -10.34 | 71.91 | 14 | 92 |
| | | 2 | -7.77 | 43.61 | 27 | |
| | | 3 | -6.73 | 27.92 | 36 | |
| | | 4 | -5.93 | 18.61 | 44 | |
| | | 5 | -4.62 | 9.28 | 55 | |
| | | 6 | -3.7 | 6.21 | 63 | |
| | | 7 | -2.68 | 3.66 | 72 | |
| | | 8 | -2.2 | 1.87 | 80 | |
| | | 9 | -1.7 | ***0.83*** | 92 | |
| 10 | 1BE9 | 1 | -10.87 | 77.03 | 10 | 49 |
| | | 2 | -9.65 | 54.18 | 27 | |
| | | 3 | -3.32 | 29.66 | 51 | |
| | | 4 | -2.09 | 22.80 | 81 | |
| | | 5 | -1.94 | 13.77 | 122 | |
| | | 6 | -1.77 | 8.77 | 158 | |
| | | 7 | -1.82 | 6.69 | 196 | |
| | | 8 | ***0.13*** | 2.89 | 233 | |

**Notes**

[1]$E_{inter}$ (intermolecular interaction energy) from the AD4 scoring function (except for System 6, where is LJ potential)

[2] Represents the free (uncovered with ligands) target surface area after each docking cycle.

[3] Nr of ligands after docking process (Wrapper process without trimming, for details, see Manual).

[4] Nr of ligands after wrapper process (includes the trimming step, for details, see Manual)

## Appendix 3

Re-ranking. Continuation and completion of the results presented at Table S8

**Results**

In the tables below, cluster representatives that started the $MD_F$ simulation with the smallest RMSD are highlighted with bold and italic.

**System 10 (1be9)**

| initial rank | final rank | $E_{LJ}$ (before minimization) | $E_{LJ}$ (after minimization) | $E_{LJ}$ (after $MD_F$) |
|---|---|---|---|---|
| 1 | 2 | -27.14 | -27.14 | -28.33 |
| ***2*** | ***1*** | ***-27.11*** | ***-29.74*** | ***-46.59*** |
| 3 | 3 | -18.69 | -15.24 | -24.32 |
| 4 | 4 | -11.37 | -13.23 | -20.31 |

**System 3 (3hvc)**

| initial rank | final rank | $E_{LJ}$ (before minimization) | $E_{LJ}$ (after minimization) | $E_{LJ}$ (after $MD_F$) |
|---|---|---|---|---|
| 1 | 1 | -30.89 | -28.2568 | -30.3297 |
| 2 | 7 | -29.90 | -23.0536 | -19.9716 |
| ***3*** | ***4*** | ***-29.59*** | ***-27.8962*** | ***-26.0048*** |
| 4 | 15 | -25.52 | -22.2557 | -10.5898 |
| 5 | 13 | -22.91 | -19.9508 | -16.6608 |
| 6 | 3 | -22.42 | -20.303 | -27.7106 |
| 7 | 11 | -20.16 | -19.3671 | -17.5456 |
| 8 | 20 | -18.60 | -17.2555 | -0.0054 |
| ***9*** | ***12*** | ***-17.71*** | ***-15.4347*** | ***-17.3324*** |
| 10 | 16 | -17.41 | -16.3628 | -10.5569 |
| 11 | 6 | -16.66 | -9.9727 | -20.7901 |
| 12 | 9 | -15.35 | -12.3445 | -19.029 |
| 13 | 19 | -14.99 | -14.9193 | -0.015 |
| 14 | 2 | -14.82 | -18.7019 | -27.7333 |
| 15 | 14 | -13.23 | -10.8594 | -11.6919 |
| 16 | 21 | -11.02 | -6.4972 | -0.0006 |
| 17 | 17 | -10.78 | -10.2724 | -10.3253 |
| 18 | 5 | -9.58 | -7.0963 | -20.9788 |
| 19 | 18 | -9.46 | -8.8948 | -8.8948 |
| 20 | 8 | -7.35 | -8.6909 | -19.2943 |
| 21 | 10 | -6.67 | -3.7734 | -18.8389 |

**System 7 (2bal)**

| initial rank | final rank | $E_{LJ}$ (before minimization) | $E_{LJ}$ (after minimization) | $E_{LJ}$ (after $MD_F$) |
|---|---|---|---|---|
| 1 | 1 | -37.25 | -43.3493 | -48.8083 |
| 2 | 6 | -35.6 | -36.0715 | -29.2095 |
| 3 | 5 | -35.6 | -35.2552 | -29.3107 |
| ***4*** | ***4*** | ***-31.98*** | ***-33.8952*** | ***-36.8991*** |
| 5 | 10 | -30.88 | -32.1657 | -18.3555 |

| | | | | |
|---|---|---|---|---|
| 6 | 2 | -27.17 | -25.3235 | -42.6772 |
| 7 | 11 | -21.85 | -21.1381 | -15.2177 |
| 8 | 3 | -16.46 | -17.5582 | -41.5161 |
| 9 | 8 | -16.17 | -17.6666 | -22.2571 |
| 10 | 12 | -15.75 | -17.6196 | -13.1258 |
| 11 | 9 | -15.58 | -17.8921 | -21.5188 |
| 12 | 7 | -14.58 | -14.6554 | -23.9522 |
| **System 8 (1hvy)** | | | | |
| **initial rank** | **final rank** | **$E_{LJ}$ (before minimization)** | **$E_{LJ}$ (after minimization)** | **$E_{LJ}$ (after $MD_F$)** |
| ***1*** | ***1*** | ***-46.6*** | ***-45.1687*** | ***-51.0286*** |
| 2 | 2 | -40.24 | -39.6745 | -29.1758 |
| 3 | 5 | -25.07 | -20.5809 | -27.0807 |
| 4 | 7 | -23.88 | -20.0736 | -23.2629 |
| 5 | 6 | -23.62 | -20.366 | -23.6756 |
| 6 | 9 | -20.27 | -20.276 | -20.2797 |
| 7 | 4 | -17.5 | -13.5324 | -27.4101 |
| 8 | 10 | -14.8 | -13.2025 | -18.6349 |
| 9 | 8 | -13.8 | -13.9297 | -21.6119 |
| 10 | 3 | -6.24 | -6.7145 | -28.5756 |
| **System 2 (3n3l)** | | | | |
| **initial rank** | **final rank** | **$E_{LJ}$ (before minimization)** | **$E_{LJ}$ (after minimization)** | **$E_{LJ}$ (after $MD_F$)** |
| 1 | 1 | -42.56 | -36.927 | -33.2926 |
| ***2*** | ***4*** | ***-29.01*** | ***-30.2467*** | ***-20.748*** |
| 3 | 3 | -27.48 | -22.3244 | -23.8049 |
| 4 | 2 | -19.84 | -17.2051 | -24.9051 |
| 5 | 7 | -18.05 | -17.7004 | -17.6639 |
| 6 | 10 | -17.77 | -18.1652 | -15.5199 |
| 7 | 13 | -17.64 | -16.3152 | -10.0326 |
| 8 | 12 | -14.51 | -12.8485 | -12.6963 |
| 9 | 8 | -14.41 | -14.68 | -17.5498 |
| 10 | 5 | -14.35 | -10.9736 | -19.3694 |
| 11 | 6 | -13.24 | -19.2486 | -19.2375 |
| 12 | 9 | -8.33 | -11.0855 | -15.9268 |
| 13 | 11 | -7.55 | -7.254 | -13.5663 |
| **System 9 (3g5d)** | | | | |
| **initial rank** | **final rank** | **$E_{LJ}$ (before minimization)** | **$E_{LJ}$ (after minimization)** | **$E_{LJ}$ (after $MD_F$)** |
| 1 | 2 | -51.01 | -53.6333 | -47.3457 |
| ***2*** | ***1*** | ***-41.55*** | ***-43.86*** | ***-48.2548*** |
| 3 | 6 | -33.88 | -33.4359 | -30.0882 |
| 4 | 5 | -32.26 | -33.5511 | -31.0492 |
| 5 | 8 | -32.21 | -29.2468 | -25.21 |

| | | | | |
|---|---|---|---|---|
| 6 | 9 | -30.19 | -30.1126 | -23.683 |
| 7 | 3 | -24.18 | -25.1898 | -35.5112 |
| 8 | 10 | -19.52 | -19.2211 | -21.4805 |
| 9 | 7 | -12.85 | -12.1188 | -27.6759 |
| 10 | 4 | -9.43 | -10.3177 | -33.1655 |
| **System 5 (1qcf)** | | | | |
| **initial rank** | **final rank** | **$E_{LJ}$ (before minimization)** | **$E_{LJ}$ (after minimization)** | **$E_{LJ}$ (after $MD_F$)** |
| 1 | 2 | -34.81 | -34.5942 | -28.2804 |
| ***2*** | ***1*** | ***-34.02*** | ***-32.6234*** | ***-38.9789*** |
| 3 | 3 | -27.54 | -28.2054 | -27.1894 |
| 4 | 5 | -26.84 | -28.4516 | -24.3687 |
| 5 | 6 | -24.46 | -23.825 | -24.108 |
| 6 | 4 | -21.17 | -21.1819 | -25.5256 |
| 7 | 8 | -17.59 | -17.1838 | -20.614 |
| 8 | 9 | -16.93 | -17.3768 | -18.3152 |
| 9 | 7 | -16.11 | -15.7602 | -22.3647 |
| 10 | 10 | -15.41 | -16.8545 | -17.0075 |
| 11 | 12 | -15.41 | -13.4218 | -11.7576 |
| 12 | 11 | -9.2 | -11.359 | -13.7187 |
| **System 1 (3ptb)** | | | | |
| **initial rank** | **final rank** | **$E_{LJ}$ (before minimization)** | **$E_{LJ}$ (after minimization)** | **$E_{LJ}$ (after $MD_F$)** |
| ***1*** | ***1*** | ***-18.42*** | ***-14.3699*** | ***-15.9513*** |
| 2 | 4 | -18.4 | -14.6544 | -11.1241 |
| 3 | 2 | -15.17 | -13.2797 | -14.7309 |
| 4 | 6 | -9.54 | -7.8168 | -0.0091 |
| 5 | 3 | -5.33 | -5.8971 | -11.2752 |
| 6 | 5 | -3.18 | -3.7247 | -3.0688 |
| **System 6 (1h61)** | | | | |
| **initial rank** | **final rank** | **$E_{LJ}$ (before minimization)** | **$E_{LJ}$ (after minimization)** | **$E_{LJ}$ (after $MD_F$)** |
| ***1*** | ***2*** | ***-41.45*** | ***-34.8026*** | ***-34.4004*** |
| 2 | 1 | -38.46 | -36.9991 | -21.6876 |
| 3 | 3 | -24.71 | -25.8953 | -26.1655 |
| 4 | 5 | -21.45 | -20.1823 | -19.1498 |
| 5 | 4 | -19.08 | -22.1524 | -17.0355 |
| 6 | 6 | -15.74 | -17.2365 | -9.2827 |
| 7 | 8 | -15.32 | -15.455 | -14.8018 |
| 8 | 9 | -14.06 | -15.3818 | -17.9953 |
| 9 | 10 | -13.51 | -10.221 | -14.776 |
| 10 | 11 | -13.05 | -9.5464 | -16.1073 |
| 11 | 7 | -10.45 | -17.0566 | -11.1185 |
| 12 | 12 | -5.01 | -4.963 | -11.2832 |

| System 4 (3cpa) | | | | |
|---|---|---|---|---|
| **initial rank** | **final rank** | **$E_{LJ}$ (before minimization)** | **$E_{LJ}$ (after minimization)** | **$E_{LJ}$ (after $MD_F$)** |
| ***1*** | ***1*** | ***-45.56*** | ***-47.1921*** | ***-44.5864*** |
| 2 | 7 | -28.47 | -26.219 | -0.0839 |
| 3 | 4 | -27.89 | -28.362 | -24.8 |
| 4 | 8 | -26.22 | -29.8702 | 0.0027 |
| 5 | 2 | -25.9 | -25.7242 | -25.3906 |
| 6 | 3 | -17.72 | -17.1598 | -25.0904 |
| 7 | 6 | -15.49 | -16.2944 | -0.2848 |
| 8 | 5 | -8.57 | -9.9116 | -10.4001 |

# Appendix 4

Re-scoring. Completion and continuation of results presented at Table S13

**Results**

Ligand poses with the smallest RMSD are highlighted with bold and italic.

System 8 (1hvy)

| Clusters | RMSD with $E_{LJ}$ score (Å) | RMSD with score AD4 (Å) |
|---|---|---|
| 1 | ***3.455*** | ***3.455*** |
| 2 | 21.374 | 21.903 |
| 3 | 18.682 | 21.374 |
| 4 | 26.487 | 10.28 |
| 5 | 30.395 | 28.356 |
| 6 | 31.837 | 30.323 |
| 7 | 24.48 | 15.818 |
| 8 | 12.892 | 24.48 |
| 9 | 31.887 | 26.989 |
| 10 | 15.818 | 31.887 |

**System 1 (3ptb)**

| Clusters | RMSD with $E_{LJ}$ score (Å) | RMSD with AD4 score (Å) |
|---|---|---|
| 1 | ***4.336*** | ***0.283*** |

**System 5 (1qcf)**

| Clusters | RMSD with $E_{LJ}$ score (Å) | RMSD with score AD4 (Å) |
|---|---|---|
| 1 | 34.063 | 27.362 |
| 2 | 14.255 | 29.069 |
| 3 | 29.127 | 11.626 |
| 4 | 28.567 | ***1.593*** |
| 5 | 28.783 | 23.398 |
| 6 | 23.372 | 34.095 |
| 7 | ***1.637*** | 27.523 |

**System 9 (3g5d)**

| Clusters | RMSD with $E_{LJ}$ score (Å) | RMSD with score AD4 (Å) |
|---|---|---|
| 1 | 22.578 | 28.673 |
| 2 | 9.322 | ***1.331*** |
| 3 | ***2.764*** | 15.223 |
| 4 | 37.059 | 24.913 |
| 5 | 27.079 | 22.755 |
| 6 | 28.669 | 34.634 |
| 7 | 28.823 | 26.826 |
| 8 | 25.355 | 28.823 |
| 9 | 22.755 | 22.493 |
| 10 | 18.327 | 22.53 |
| 11 | 24.035 | 25.355 |
| 12 | 16.21 | 16.21 |
| 13 | 22.493 | 19.466 |
| 14 | 19.466 | 35.487 |

| | | |
|---|---|---|
| 15 | 31.288 | |
| 16 | 35.487 | |
| **System 7 (2bal)** | | |
| **Clusters** | **RMSD with $E_{LJ}$ score (Å)** | **RMSD with score AD4 (Å)** |
| 1 | 27.593 | 18.373 |
| 2 | 21.583 | 25.58 |
| 3 | ***9.973*** | 14.436 |
| 4 | 17.076 | 17.076 |
| 5 | 23.954 | 37.498 |
| 6 | 17.413 | 29.474 |
| 7 | 17.941 | ***3.945*** |
| 8 | 26.057 | 17.84 |
| 9 | 10.434 | 18.07 |
| 10 | 28.775 | 22.668 |
| 11 | 27.61 | 27.589 |
| 12 | 37.767 | 27.593 |
| 13 | 22.668 | 27.61 |
| 14 | 27.582 | 28.676 |
| 15 | 30.291 | |
| 16 | 28.676 | |
| **System 4 (3cpa)** | | |
| **Clusters** | **RMSD with $E_{LJ}$ score (Å)** | **RMSD with score AD4 (Å)** |
| 1 | ***3.377*** | ***3.377*** |
| 2 | 22.657 | 20.833 |
| 3 | 31.073 | 28.515 |
| 4 | 30.712 | 30.712 |
| 5 | 28.325 | 23.627 |
| 6 | 31.752 | 18.239 |
| 7 | 23.627 | 28.782 |
| 8 | 18.239 | 19.101 |
| 9 | 26.42 | 16.823 |
| 10 | 25.706 | 21.423 |
| 11 | 16.27 | 31.752 |
| 12 | 21.423 | 23.367 |
| 13 | 28.752 | 20.795 |
| 14 | 21.444 | 21.51 |
| 15 | 20.795 | 21.444 |
| 16 | 35.714 | 36.145 |
| 17 | 16.989 | 28.325 |
| **System 3 (3hvc)** | | |

| Clusters | System 3a RMSD (Å) with $E_{LJ}$ score | System 3b RMSD (Å) with $E_{LJ}$ score | System 3a RMSD (Å) with AD4 score | System 3b RMSD with AD4 score |
|---|---|---|---|---|
| 1 | 15.288 | 31.353 | 14.147 | 44.341 |
| 2 | ***8.113*** | 31.369 | 15.999 | 32.151 |
| 3 | 16.939 | 26.37 | 18.827 | 25.873 |
| 4 | 27.24 | 35.058 | ***8.898*** | 31.325 |
| 5 | 36.556 | ***20.142*** | 37.243 | 19.844 |
| 6 | 15.101 | 44.356 | 19.497 | 49.933 |
| 7 | 19.561 | 49.933 | 27.032 | 35.058 |
| 8 | 31.358 | 23.771 | 31.488 | ***23.771*** |

**System 10 (1be9)**

| Clusters | RMSD with $E_{LJ}$ score (Å) | RMSD with score AD4 (Å) |
|---|---|---|
| 1 | 19.962 | ***6.598*** |
| 2 | 24.511 | 21.35 |
| 3 | 30.323 | 25.101 |
| 4 | ***7.25*** | 18.302 |
| 5 | 27.04 | 21.719 |
| 6 | 21.068 | 32.616 |
| 7 | 11.905 | 10.41 |
| 8 | 10.41 | 21.014 |
| 9 | 18.827 | 23.854 |
| 10 | 24.927 | 22.547 |
| 11 | 22.547 | |
| 12 | 21.014 | |
| 13 | 23.854 | |

**System 6 (1h61)**

| Clusters | RMSD with $E_{LJ}$ score (Å) | RMSD with score AD4 (Å) |
|---|---|---|
| 1 | ***3.461*** | ***6.756*** |
| 2 | 31.24 | 31.943 |
| 3 | 33.031 | 27.264 |
| 4 | 26.946 | 33.109 |
| 5 | 32.364 | 27.977 |
| 6 | 28.496 | 30.992 |
| 7 | 20.168 | 20.506 |
| 8 | 31.02 | 31.087 |

**System 2 (3n3l)**

| Clusters | RMSD with $E_{LJ}$ score (Å) | RMSD with score AD4 (Å) |
|---|---|---|
| 1 | 9.664 | ***3.925*** |
| 2 | ***3.828*** | 25.65 |
| 3 | 18.018 | 9.933 |
| 4 | 25.947 | 17.81 |
| 5 | 21.246 | 30.595 |
| 6 | 30.595 | 21.246 |

## Supporting Movie Caption 1

The process of Wrapper and Shaker in case of System 5. The first part presents the results of 15 wrapping cycles. The second part captures the $MD_B$ and the two $MD_{BSA}$ cycles of Shaker. Final cluster representatives are the outputs of the WnS. Additional refinement steps are featured in Movie 2.

## Supporting Movie Caption 2

Conformational changes of pentapeptide KQTSV, bound to PDZ-domain (System 10) during 65 ns simulations performed Shaker. The binding pocket of KQTSV on the PDZ domain is presented with grey surface. The simulated and crystallographic reference structures of KQTSV are presented as teal and red sticks.

# References


Abraham, M.J., *et al.* (2015) GROMACS: High performance molecular simulations through multi-level parallelism from laptops to supercomputers, *SoftwareX*, **1**, 19-25.

Dror, R.O., *et al.* (2011) Pathway and mechanism of drug binding to G-protein-coupled receptors, *P Natl Acad Sci USA*, **108**, 13118-13123.

Frisch, M., *et al.* (2009) Gaussian 09, revision D. 01. Gaussian, Inc., Wallingford CT.

Guex, N. and Peitsch, M.C. (1997) SWISS-MODEL and the Swiss-Pdb Viewer: an environment for comparative protein modeling, *Electrophoresis*, **18**, 2714-2723.

Hetenyi, C. and van der Spoel, D. (2006) Blind docking of drug-sized compounds to proteins with up to a thousand residues, *Febs Letters*, **580**, 1447-1450.

Hetenyi, C. and van der Spoel, D. (2011) Toward prediction of functional protein pockets using blind docking and pocket search algorithms, *Protein Sci*, **20**, 880-893.

Jorgensen, W.L., *et al.* (1983) Comparison of simple potential functions for simulating liquid water, *J Chem Phys*, **79**, 926-935.

Krishnan, R., Frisch, M. and Pople, J. (1980) Contribution of triple substitutions to the electron correlation energy in fourth order perturbation theory, *J Chem Phys*, **72**, 4244-4245.

Lindorff-Larsen, K., *et al.* (2010) Improved side-chain torsion potentials for the Amber ff99SB protein force field, *Proteins: Struct, Funct, Bioinf,* **78**, 1950-1958.

MOPAC, S.J. (2012) Stewart computational chemistry. Version.

Morris, G.M., *et al.* (2009) AutoDock4 and AutoDockTools4: Automated Docking with Selective Receptor Flexibility, *J Com Chem*, **30**, 2785-2791.

Release, S. (2013) 3: Maestro, version 9.6, *Schrödinger, LLC, New York, NY*.

Shan, Y.B., *et al.* (2011) How Does a Drug Molecule Find Its Target Binding Site?, *J Am Chem Soc*, **133**, 9181-9183.

Tiwary, P., *et al.* (2015) Kinetics of protein–ligand unbinding: Predicting pathways, rates, and rate-limiting steps, *P Natl Acad Sci USA*,, **112**, E386-E391.

Vanquelef, E., *et al.* (2011) RED Server: a web service for deriving RESP and ESP charges and building force field libraries for new molecules and molecular fragments, *Nucleic Acids Res*, **39**, W511-W517.

Wang, J., *et al.* (2012) Development of Polarizable Models for Molecular Mechanical Calculations. 3. Polarizable Water Models Conforming to Thole Polarization Screening Schemes, *J. Phys. Chem. B*, **116**, 7999-8008.
